# Quantification of the neuropathology of alcohol use disorder using tissue microarrays

**DOI:** 10.1093/jnen/nlaf147

**Published:** 2026-03-01

**Authors:** Jie Liu, Matthew J. Catanzariti, Huang-Tuong Nguyen-Hao, Mario Novelli, Dhiraj Maskey, Caine C. Smith, Julia C. Stevens, Markus J. Hofer, Suzanne M. de la Monte, Greg Trevor Sutherland

**Affiliations:** 1 Charles Perkins Centre and School of Medical Sciences, Faculty of Medicine and Health, The University of Sydney, Camperdown, NSW, Australia; 2 School of Life and Environmental Sciences and Charles Perkins Centre, Faculty of Science, The University of Sydney, Sydney, NSW, Australia; 3 Departments of Pathology and Laboratory Medicine, Neurology, Neurosurgery, and Medicine, Rhode Island Hospital, Brown University Health, Alpert Medical School of Brown University, Providence, RI, United States; 4 Department of Medicine, Rhode Island Hospital, Lifespan Academic Institutions, and the Warren Alpert Medical School of Brown University, Providence, RI, United States

**Keywords:** alcohol use disorder, immunohistochemistry, neuropathology, postmortem brain tissue, tissue microarray

## Abstract

Alcohol use disorder (AUD) is characterized by an inability to stop consuming alcohol. Neuroimaging studies of patients with AUD show mild grey and white matter atrophy, while pathological studies suggest that atrophy is restricted to the white matter. The effects on individual brain cells are largely unknown. Mild neuronal loss has been described in the prefrontal cortex but this has not been consistent. Studies quantifying oligodendrocytes, astrocytes, and microglia are rare. These knowledge gaps impede therapeutic advancements. Here, we piloted the use of tissue microarrays, immunohistochemistry, and automated image analysis to systematically quantify cell profiles in human postmortem tissue. We sampled 173 grey and white matter cores across 5 cerebral regions from 4 male AUD cases and 4 age-matched controls. We found no obvious differences in the regional profiles of neurons, astrocytes, oligodendrocytes, or microglia. However, mean microglial densities across all regions were higher in AUD (*P* =.0024). There were visual signs of reactive astrocytosis in AUD cases but mean cell body sizes were unchanged. Our findings suggest that alcohol-related brain damage is not due to a loss of any of major cell classes. Larger studies focusing on subtype-specific markers and advanced image analysis tools are required.

## INTRODUCTION

Alcohol use disorder (AUD) is a psychiatric disorder diagnosed through behavioral, cognitive, and physiological criteria with chronic alcohol misuse being a key feature. Chronic alcohol misuse can lead to changes in brain structure and chemistry. These changes undermine addiction and increase the susceptibility of individuals to relapse. In some individuals, this pattern of chronic alcohol misuse is also neurodegenerative, that is, characterized by progressive loss of working memory and attention. The combination of addictive and neurodegenerative effects is referred to as alcohol-related brain injury or damage (ARBD).

Unlike neurodegenerative disorders, such as Alzheimer disease (AD), there are no hallmark pathologies in ARBD. Previous pathological studies have shown mild neuronal loss in the prefrontal cortex (PFC), while loss in other regions, such as the cerebellum was seen only in those individuals with concomitant Wernicke encephalopathy.^1–6^ Consistent with the relative lack of neuronal loss in uncomplicated AUD cases, volumetric studies of postmortem tissue in patients with AUD have shown widespread white matter (WM) loss but not grey matter (GM) atrophy.^7,8^ The latter studies contrast with neuroimaging studies in which atrophy of select GM regions has been seen; both modalities show WM damage.^9^ Interestingly for a neurodegenerative disease, neuroimaging studies have repetitively shown that abstinence results in a partial recovery of WM. The mechanisms of WM loss and recovery in ARBD may be generalizable to other brain diseases, providing wider therapeutic opportunities.

Neurons, specifically those in the cerebellum, hippocampus, and PFC, are generally regarded as the brain cells most sensitive to chronic alcohol intoxication,^10^ with PFC neuron loss also linked to addictive behavior.^11^ This represents a potential disconnect with the more dominant finding of WM atrophy.^7,12^ WM damage could be secondary to neuron loss but ARBD may also be a primary gliopathy. Microglia and astrocytes have both been implicated in the pathogenesis of ARBD. Oligodendrocytes, however, in view of with their greater density in WM compared to GM,^13^ and known myelin protein deficits in ARBD,^14,15^ represent the most obvious glial cell for further investigation.

We have previously used MALDI imaging^16^ and mass spectrometry to show that myelin lipids are modified in AUD postmortem tissue.^17^ Specifically, decreases in phospholipids and ceramide were seen in both the GM and WM, including the PFC but also in the visual cortex, which was not known to be affected by ARBD. It is unclear whether this represents changes in the oligodendrocyte density, subtypes, or lipid metabolism.

Stereological approaches are the gold standard for quantifying cells and pathology in the human brain.^18^ However, the resource-intensive nature of these approaches and the size of the human brain have meant that global and quantitative neuropathological assays of ARBD have not been undertaken. A related issue is that automated image analysis software, which is commonly employed in quantification of disease models, is yet to be fully validated in postmortem human brain tissues.

The lack of a neuropathological correlate in ARBD creates a fundamental gap in our understanding of how individuals and brain regions are relatively susceptible or resistant to the effects of alcohol. This is an essential requirement for identifying therapeutic targets for AUD. For an organ the size of the human brain, there needs to be trade-off between breadth and depth of cell quantitation studies. Tissue microarrays (TMA) offer a high-throughput solution to assay single targets across large cohorts.^19^ Alternatively, they can be employed to assay multiple sites in a single individual. Martikainen et al^20^ demonstrated the latter when showing the cross-over of hallmark pathologies between 5 subjects with different neurodegenerative diseases. We recently combined TMAs with multiplex immunofluorescence (mIF) to demonstrate AD pathologies and their spatial relationships with the major classes of brain cells.^21^ We predict that regional susceptibility will manifest in the loss of one of these major cell types in ARBD.

Here, we combined TMAs with a semi-automated image analysis workflow to quantify neurons and non-neuronal cells in a total of 173 GM and WM cores across 4 individuals with AUD and 4 age- and gender-matched controls. This “first pass” study gives a novel helicopter view of regional and cell-specific susceptibility to chronic alcohol intoxication across the human cerebrum.

## METHODS

### Tissue acquisition

Formalin-fixed and paraffin-embedded tissue blocks from 4 male AUD cases and 4 age- and gender-matched controls were provided by the New South Wales Brain Tissue Research Centre (BTRC), following approval from their Scientific Advisory Committee and the University of Sydney Human Research Ethics Committee (2018/HE000477) (Table 1). The BTRC provided AUD status (DSM-V) an AUDIT-C score, lifetime alcohol consumption information, cause of death, liver pathology, brain pH, and postmortem interval, as previously described.^22^ This proof-of-concept study was restricted to males. Standard tissue blocks had been fixed for 3 weeks prior to processing and paraffin embedding. Pathological assessments were carried out by an experienced BTRC neuropathologist to exclude other neurological (neurodegenerative) diseases. The BTRC takes 16 standard blocks from each fixed hemisphere for diagnostic purposes and as the commonly requested regions for research.^22^ Here, 5 blocks were chosen to represent a broad selection of brain regions, specifically those previously shown to be affected by neuronal loss in AUD.^4^ TMA design and construction have been described previously.^21^ Briefly each TMA was constructed with 16 × 2 mm cores made up of 5 neocortical regions: PFC (4 cores; 2 GM cores, an adjacent WM core, and a deep WM core), superior parietal cortex ([SPC]; 3 cores; 2 GM cores, 1 adjacent WM core), primary visual cortex ([PVC]; 3 cores; 2 GM cores, 1 adjacent WM core), superior temporal gyrus ([STG]; 3 cores; 2 GM cores, 1 adjacent WM core), and anterior cingulate cortex ([ACC]; 3 cores; 1 GM core, 1 adjacent WM core, 1 corpus callosum [CC] core). Subcortical regions including the entorhinal cortex, cerebellum, and pons were also sampled but were not analyzed here. TMA blocks were subsequently annealed using 4 cycles of 1 hour heating at 37 °C and 1 hour at 4 °C. Finally, blocks were incubated at 46 °C for 1 hour, followed by rehydration in a water bath before sectioning (7 μm) for mIF; using a HM325 Manual Microtome (ThermoScientific).

### Immunohistochemistry

TMA slides were initially dewaxed and rehydrated via serial solutions of alcohol. Slides were additionally incubated with 10% neutral buffered formalin for 10 minutes to improve tissue adhesion. Non-specific heat-induced epitope retrieval (HIER) was performed by pressure cooking (Decloaking Chamber NxGen, Biocare Medical) in AR6 Buffer (Akoya Biosciences, Marlborough, MA, United States) at 110 °C and 5 psi for 10 minutes. Adjacent 7-μm TMA sections were individually stained overnight at 4 °C with the following primary antibodies: anti-NeuN antibody (rabbi; 1:1000, Merck, Cat. No. ABN78), anti-ASPA (rabbit; 1:1000; Agilent, Cat. No. Ab223269) anti-ALDH1L1 (mouse; 1:1000; UltraLab, Cat. No. UM570040), anti-GFAP (rabbit; 1:2000; Agilent, Cat. No. Z033401); anti-AQP4 (rabbit; 1:2000; Sigma, Cat. No. A5971) and anti-Iba1 (rabbit; 1:1000; Wako Pure Chemical Industries, Cat. No. 019–1974). HIER-treated slides were sequentially treated for endogenous peroxidase and non-specific protein binding with 3% hydrogen peroxide in methanol and 10% normal horse serum (Gibco, Lot No. 2054010) for 10 minutes each. Marker visualization was carried out with 3,3′-diaminobenzidine (DAB) and counterstained with hematoxylin.

### Multiplex immunofluorescence

mIF was performed on glial cell markers along with the proliferative marker, Ki-67 (mouse; 1:500; Dako, Cat. No. M7240) and 4′,6-diamidino-2-phenylindole (DAPI). As a positive control, 7-μm sections from a 62-year-old female with a stage IV glioblastoma were used (supplied by BTRC). mIF was performed according to the tyramide substrate amplification protocol from Akoya Bioscience. In brief, target proteins were labeled by sequential incubations with antigen-specific primary antibodies, anti-mouse/rabbit secondary antibody, and finally Opal tyramide fluorophores. HIER was performed for 10 minutes between each target of interest to remove the bound primary and secondary antibodies, but not the fluorophore. Astrocytes and microglia were sequentially labeled using anti-GFAP, anti-IBA1 anti-ASPA, anti-ALDH1L1, anti-AQP4, and anti-Ki-67. Primary antibodies were detected using Anti-Ms/Rb HRP secondary antibody and Opal 690, 520, 480, 570, 620, and 780, respectively. Following the final HIER stripping, slides were incubated with DAPI and mounted with ProLong Diamond Antifade mounting agent (Invitrogen, Thermo Fisher Scientific).

### Image capture and analysis

Image acquisitions for both mIF and chromogenic slides were carried out using the PhenoImager Fusion (Akoya Biosciences) at 20× magnification and a resolution of 0.50 μm/pixel. Focal points were automated, and exposures for each channel were individually assessed for each TMA to account for variability in staining quality. Post-scanning, images were further controlled for autofluorescence and signal crosstalk with inForm (Akoya Bioscience) using a custom Opal monoplex and brain-specific autofluorescence library.

Image analyses were conducted in QuPath (version 0.5.1) using its proprietary image analysis tools.^23^ Manual TMA detection was performed using the “Ellipse Annotation” in conjunction with the “Brush” tool to correct for staining artifacts and tissue damage. The perimeter of cores was excluded in these ROIs to avoid “edge” effects (non-specific tissue-boundary staining). Cell quantifications, other than microglia, were automated using DAPI signals with QuPath’s “Cell Detection” function. Initially, manual counts were performed with a 22 500 μm^2^ ROI to determine pixel intensity thresholding for cores from each region. Then, iterative automated quantification to match manual counts allowed the thresholding value to be extrapolated to the ROI of these cores.^24^ The sigma values for the Gaussian filter and minimum nuclei area were additionally increased to 2 and 10 μm^2^, respectively to minimize fragmentation of euchromatic nuclei while all other parameters were defaulted. Classification of cell types was performed with QuPath’s “Single Measurement Classifier” tool. Given the highly ramified morphology of glia, positive cells were classified only if their fluorescence signal colocalized with a DAPI-segmented cell body. This method minimized false positives, such as where fragmented processes overlap the nuclei of other cell types. Thresholding these fluorescence signals was specific to the standard block from which the cores were derived. For immunohistochemistry, automated NeuN^+^ cell detection was achieved with DAB optical density using a similar manual and automated count-matching technique to determine thresholds. In both mIF and chromogenic analyses, automated counts and classifications were manually checked for accuracy. Quantification of pathologies and cell types was subsequently performed on an average of values across the 2 cores from the same standard block for each individual.

For microglial quantification, with their range of morphologies, an alternative semi-automated counting method was used. This involved training a deep learning object detection model known as “Fast R-CNN”.^25^ Ground truth annotations were manually created using V7 Darwin (darwin.v7labs.com), where bounding boxes were drawn around nuclei and microglia. A nucleus was marked with a bounding box if clearly demarcated hematoxylin was present in a shape that resembled a nucleus. Microglia were marked if they had a healthy, reactive, amoeboid, or dystrophic morphology. In total, 449 images were manually annotated, with 7102 nuclei and 696 microglia annotated. Annotations were exported in the COCO JSON format. The model was trained using Google Colab, with an Nvidia T4 Tensor Core GPU, 13 GB of usable RAM, and 15 GB of usable GRAM. The “detectron2” Python library was used for training.^26^ The model was trained over 5000 iterations with a base learning rate of 0.00025. A focal loss function was used, with an alpha value of 0.25, and a gamma value of 2. A confidence threshold of 0.2 was used for predictions. Whole section images (WSIs) were then analyzed by the model, and bounding boxes were automatically placed over structures that exceeded the set confidence threshold. Rectangular ROIs were then manually selected from the annotated output, and microglia density was calculated. This alternative method was highly correlated with the QuPath-based approach above but was adopted here as it dealt with variable morphologies more consistently during the training process.

To determine astrocyte size, 10 ALDH1L1+ astrocytes were randomly selected from each core of all individual (~200 astrocytes). Using QuPath, after appropriate thresholding, a line was manually drawn along the longest length of these astrocyte cell bodies, and the mean diameter was recorded.

### Comparison with whole-section images

WSIs were stained using the antibody panels above and sequentially paired with Opal 520 (IBA1), 570 (ALDH1L1), 480 (ASPA), and 620 (NeuN) prior to DAPI nuclear labeling. Images were captured using the Phenocycler Fusion (Akoya Bioscience) at 20× and underwent spectral unmixing with InForm to subtract autofluorescence signals.

Cell quantification was performed as per above using the Qu-Path approach. In brief, ROIs were selected using the “brush” tool to contain both a parallel cortical strip and uniform staining quality. Total cell counts were performed using DAPI signal and thresholded to enable the detection of all nuclei. This was followed by manual quality control (QC) to correct over- and under-segmentation of euchromatic nuclei. Cell classifications were done via “single measurement classifier” by using the mean fluorescence nucleus signal of each channel to determine cell type. Cell classifications were applied sequentially and followed by visual QC to correct false positive- and negative identifications. Three ROIs were quantified for each WSI (exception of 1 case due to poor tissue quality), yielding a minimum total area of analysis of 14.5 mm^2^. In addition, a heatmap of IBA1^+^ distribution was generated using QuPath “density map” using raw counts of positive IBA1 classification for each ROI and a density radius of 169.3.

### Statistical analysis

Notwithstanding the small sample size in this study, potential data trends between case and control tissues were explored using GraphPad Prism (version 10.4.1). Global comparisons were made using a Wilcoxon test, while region-by-region comparisons were made using unpaired, non-parametric Mann-Whitney tests. Family-wise correction was not performed due to the small sample size. Spearman’s coefficient was derived for correlation analyses. Composite TMA and WSI cell counts were also compared using Spearman’s coefficient.

## RESULTS

### Demographics and clinical information

Our aim was to demonstrate that TMAs can be used to capture “global” neuropathological data on ARBD from human postmortem brain tissue. The predicted outputs were regions, and cell types that displayed differing vulnerabilities, that could be selected for comparison in larger, more focused studies. In this study, we concentrated on the cerebrum because of cytoarchitectural similarities between the different areas including regions that had previously been shown to be affected (eg, PFC) or not affected (eg, PVC) by ARBD. TMAs were constructed by the BTRC, following approval from the University of Sydney Human Research Ethics Committee (2021/HE000857), and the BTRC’s Scientific Advisory Board, from 4 male AUD cases and 4 age- and gender-matched controls (Table 1). Three cases met the DSM-V criteria for severe AUD, and one case (A1) met the criteria for mild AUD. AUD cases had greater lifetime alcohol consumption than controls but individual levels were variable. All 4 cases had the maximum AUDIT-C score of 12. There were no differences in mean brain weight, postmortem interval, or brain pH between cases and controls (Table 1). A full summary of clinical and demographic data is provided in Supplementary File 1.

### Cell quantification

Each TMA contained 16, 2-mm-wide cores from the PFC (4 cores), SPC, PVC, STG, and ACC (3 cores). Each region had 2 overlapping GM cores, and 1 adjacent WM core. The exceptions were the PFC, which also had a deep WM core, and the ACC which had 1 GM, 1 WM, and 1 CC core. Adjacent 7-μm TMA sections were first stained with hematoxylin and eosin and Luxol fast blue to clearly different GM from WM and the GM-WM boundary. Further sections were stained individually for cell-specific markers: NeuN (aka RBFOX3; neurons), ASPA (oligodendrocytes), ALDHL1L, GFAP, and AQP4 (astrocytes), and Iba1 (microglia) from AUD cases (*n* = 4) and controls (*n* = 4). A summary of all stains for the 4 AUD cases and 4 controls is provided in Supplementary File 2.

### Total cell counts

**Figure 1.**
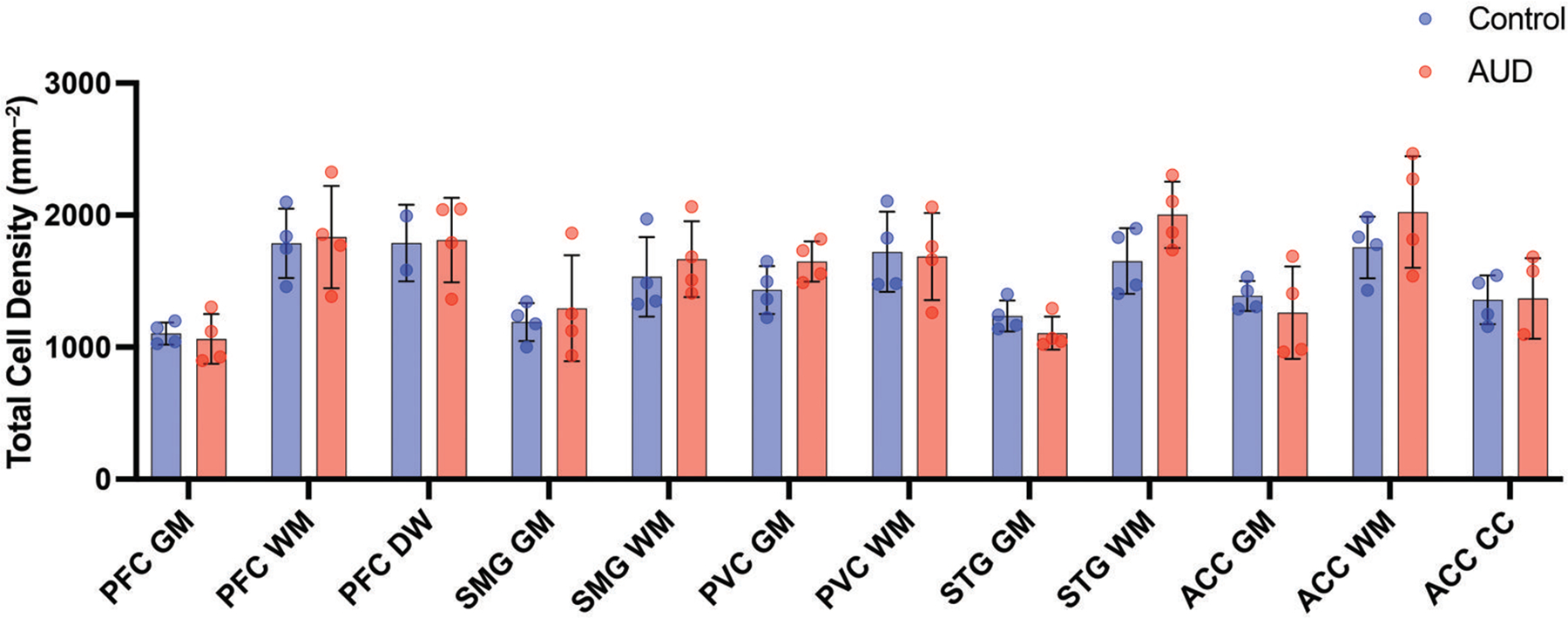
Total cell density. A histogram shows the total cell density (hematoxylin-positive cell nuclei per mm^2^) in 2 mm cores of grey matter (GM) and white matter (WM) from 5 cortical regions of 4 male alcohol use disorder (AUD, red) cases and 4 male controls (blue). Prefrontal cortex (PFC); superior parietal cortex (SPC); primary visual cortex (PVC); superior temporal gyrus (STG), anterior cingulate cortex (ACC), including corpus callosum (ACC CC). Data shown as mean ± SD.

Total cell nuclei were enumerated across all cores by training a QuPath model on the hematoxylin counterstain from the NeuN-stained sections. Cell density varied between 1000 and 1300 cells per mm^2^ in the GM, and 1200 and 1700 per mm^2^ in the WM (Figure 1). There were no differences in global total cell density between AUD cases (mean = 1564 ± 140 per mm^2^) and controls (1421 ± 156 per mm^2^; *P* = .22).

### Neuron counts

**Figure 2.**
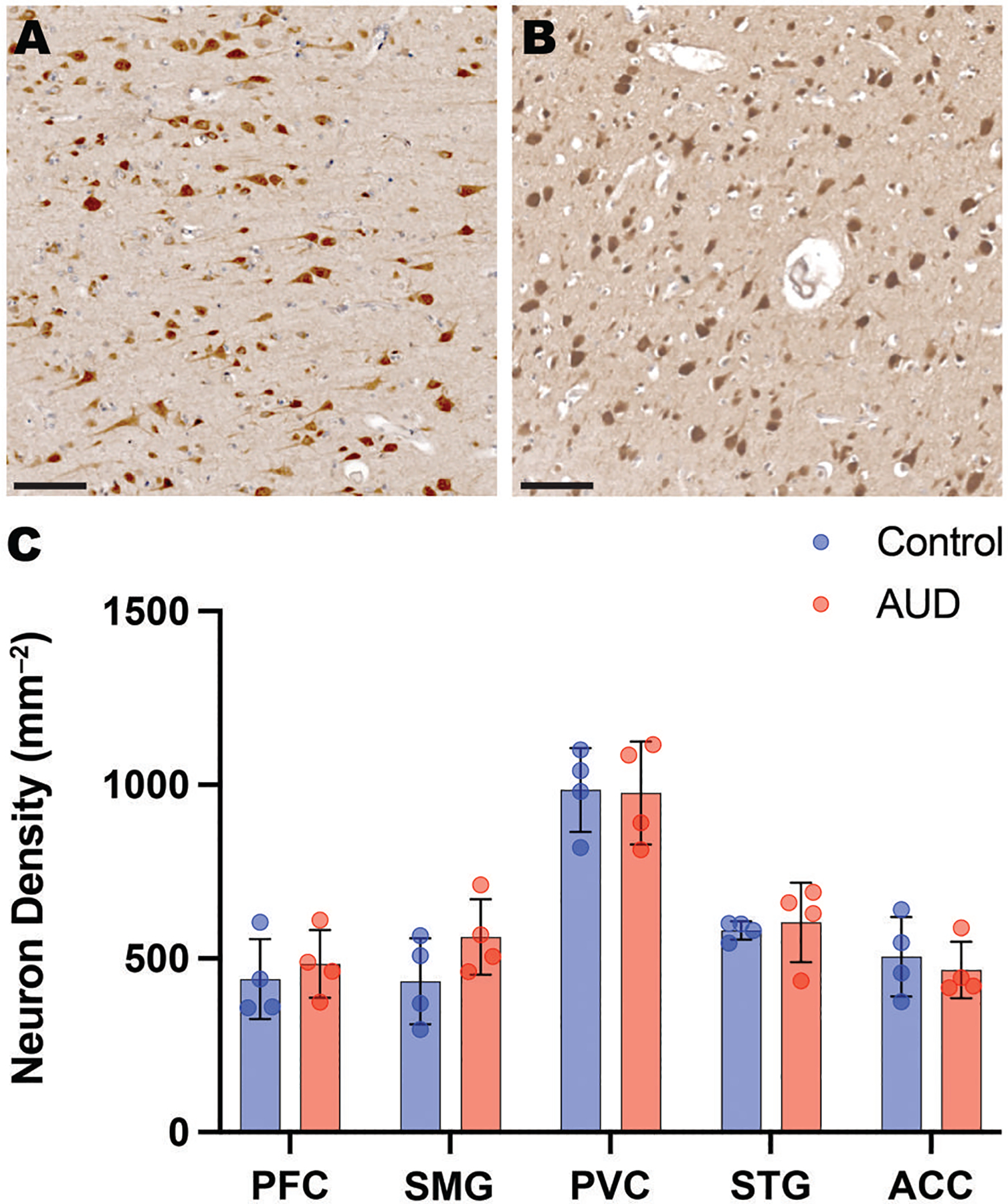
Neuron density. Neurons were quantified in 2 mm grey matter cores from 5 cortical regions of 4 male AUD cases and 4 male controls using the pan-neuronal marker, NeuN. Photomicrographs show NeuN-positive cells in (A) Control-PFC and (B) AUD-PFC. (C) A histogram shows NeuN+ cell density in the 5 cerebral regions in the 4 AUD cases (red) and 4 controls (blue). The PVC has almost twice the neuron density of the other 4 regions. Prefrontal cortex (PFC); superior parietal cortex (SPC); primary visual cortex (PVC); superior temporal gyrus (STG), anterior cingulate cortex (ACC). Scale bars = 100 μm. Data shown as mean ± SD.

Our laboratory had previously reported a moderate neuronal loss in the PFC in “uncomplicated” AUD cases.^4^ NeuN labeled all neuronal cell types throughout the cortical lamina of controls (Figure 2A) and AUD cases (Figure 2B). NeuN^+^ cell counts varied across regions but did not differ globally between AUD cases (mean = 619 ± 51 per mm^2^) and controls (589 ± 32 per mm^2^, *P* = .36) (Figure 2C). Specifically, there was no difference in the PFC of AUD cases (mean = 485±97 per mm^2^) and controls (441± 115 per mm^2^, *P* = .58), or any of the other 4 cortical regions (Figure 2C). Maximum neuron density was seen in the PVC (mean = 981 per mm^2^), followed by the STG (592 per mm^2^), SPC (498 per mm^2^), ACC (486 per mm^2^), and PFC (463 per mm^2^). There was an apparent decrease in neuron size in the AUD cases, but this was not quantified.

### Oligodendrocyte density

**Figure 3.**
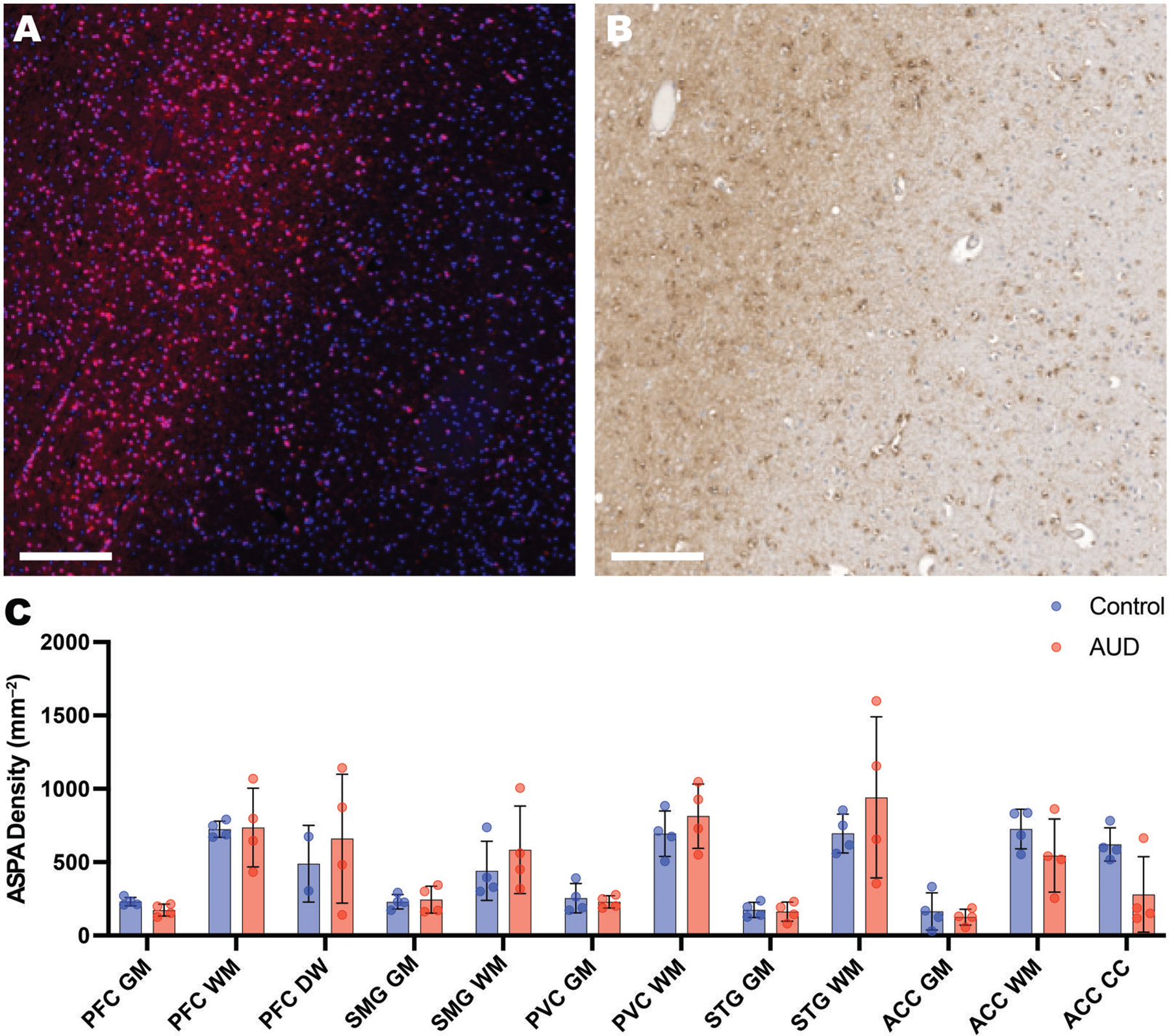
Distribution of mature oligodendrocytes at the white matter-grey matter boundary of the human cortex. Photomicrographs from adjacent sections show (A) immunofluorescent and (B) chromogenic (DAB) staining of the mature oligodendrocyte marker, aspartyl aspartate (ASPA) in the (A) occipital cortex (OC) of a 61-year-old man AUD case (A2) and (B) the prefrontal cortex (PFC) of 54-year-old male control (C4). Each image shows the higher density in white matter (left sides) vs grey matter (right sides). The immunofluorescence image shows both ASPA (pink) and the nuclear stain, DAPI (blue). (C) A histogram shows the density of oligodendrocytes quantified from 2 mm cores across 5 cortical regions of 4 male AUD cases (red) and 4 male controls (blue) using the ASPA marker. Oligodendrocytes were twice as numerous in the white matter (WM) than in the grey matter (GM). Prefrontal cortex (PFC); superior parietal cortex (SPC); primary visual cortex (PVC); superior temporal gyrus (STG), anterior cingulate cortex (ACC). Scale bars = 200 μm. Data shown as mean ± SD.

We had previously shown that AUD is characterized by widespread WM atrophy,^7,8^ with changes in the myelin lipid content,^16,17^ leading to our hypothesis that there would be a decrease in oligodendrocytes in the AUD cases. Mature oligodendrocytes were stained for ASPA^27^ by chromogenic and immunofluorescent staining and represented ~15% of total cells in the GM and 20% in WM (Figure 3). Opposing our hypothesis, there was no global difference in oligodendrocytes between AUD cases (mean = 458 ± 167 per mm^2^) and controls (433 ± 58 per mm^2^, *P* = .79). There was a trend for decreased oligodendrocytes in the CC of AUD cases (mean- = 279± 257 per mm^2^) and controls (621±113 per mm^2^, *P* = .05) and the PFC GM of AUD cases (mean = 172 ±41 per mm^2^) and controls (231± 28 per mm^2^, *P* = .05). While this suggests regional susceptibility, A2 was an outlier in these 2 regions, with densities more like controls.

### Microglial density

**Figure 4.**
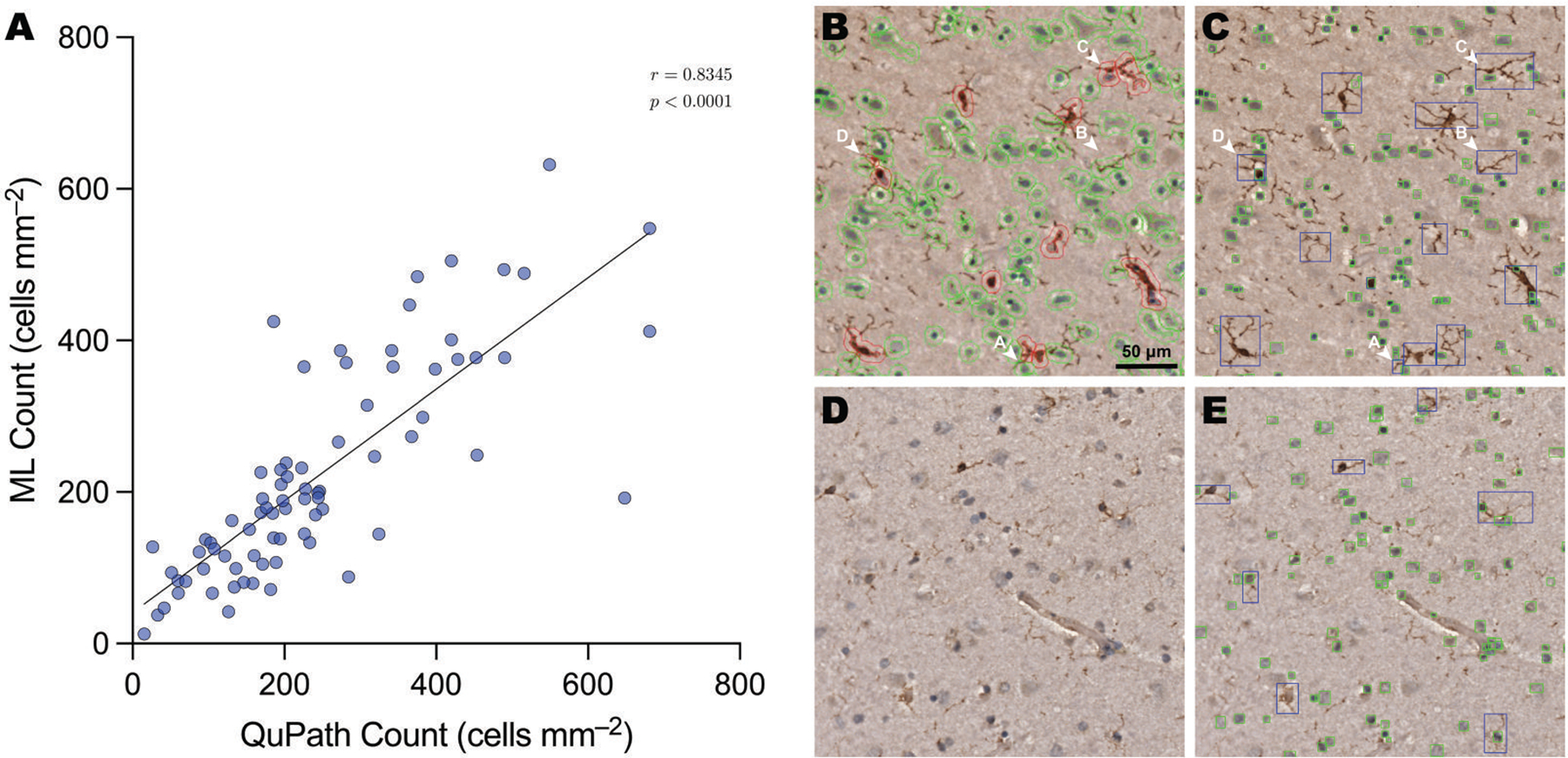
Validation of automated microglia counting against provisional QuPath counts. (A) A scatter plot showing strong alignment (*r* =0.8345; *P* < .0001) between the QuPath counts and the automated machine learning (ML)-based counts. This agreement between the methods is supported by visual examination of sample predictions. A QuPath count output (B) is compared to an ML count output (C) image. While the ML method did, at times, double-count some microglia (arrow A) and occasionally pick up fragments (arrow B), it avoided double-counting more fragmented microglia (arrows C and D), to which the QuPath method was susceptible. A faintly stained photomicrograph (D), to which QuPath had struggled to quantify, is accurately handled by the ML method (E).

We had previously shown microglial proliferation in the cau-date nucleus of AUD cases with concomitant hepatic encephalopathy but not in so-called “uncomplicated” AUD cases.^28^ Here, we expanded these studies across the entire cortex of 4 AUD cases who all had cirrhosis but only mild hepatic encephalopathy. As noted above, an alternative method of quantification was used for microglia due to their variable morphology and staining quality. The automated method of counting was provisionally validated against the QuPath-based method used for all other counts. There was strong alignment with the QuPath method (Figure 4A), with the automated method generally avoiding the double counting to which QuPath was prone (Figure 4B and C). The automated method also handled faintly stained tissue better (Figure 4D and E).

**Figure 5.**
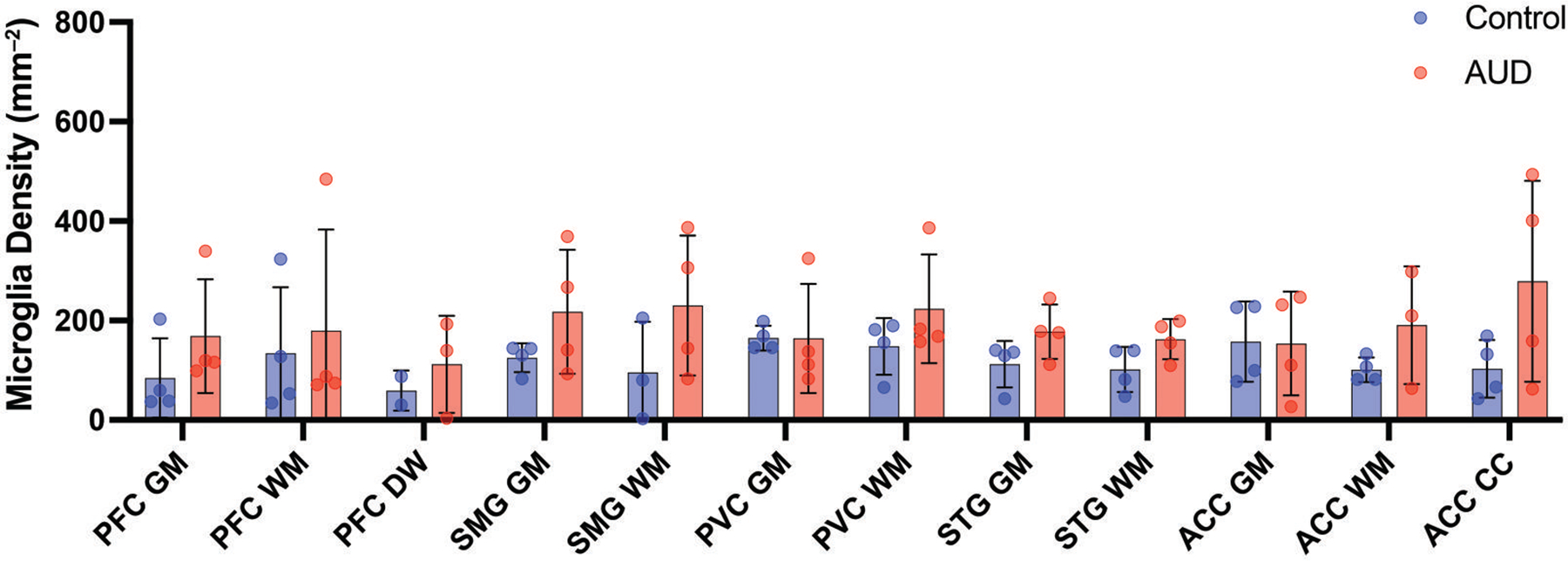
Microglial density across the human cortex of AUD cases and controls. A histogram shows the density of Iba1-immunopositive cells in grey matter (GM) and white matter (WM) of 5 cortical regions from AUD cases (red) and controls (blue). Data shown as mean ± SD.

The counts were varied but the mean IBA1^+^ cell density across all 5 cortical regions was higher in AUD cases (Figure 5; mean = 190 ± 119 per mm^2^) compared to controls (119 ± 65 per mm^2^; *P* = .0024). However, there were no differences in any specific region. Overall, the microglial fraction ranged from 6% to 18% of the total number of cells (all DAPI-nuclei) detected.

### Microglial morphology

**Figure 6.**
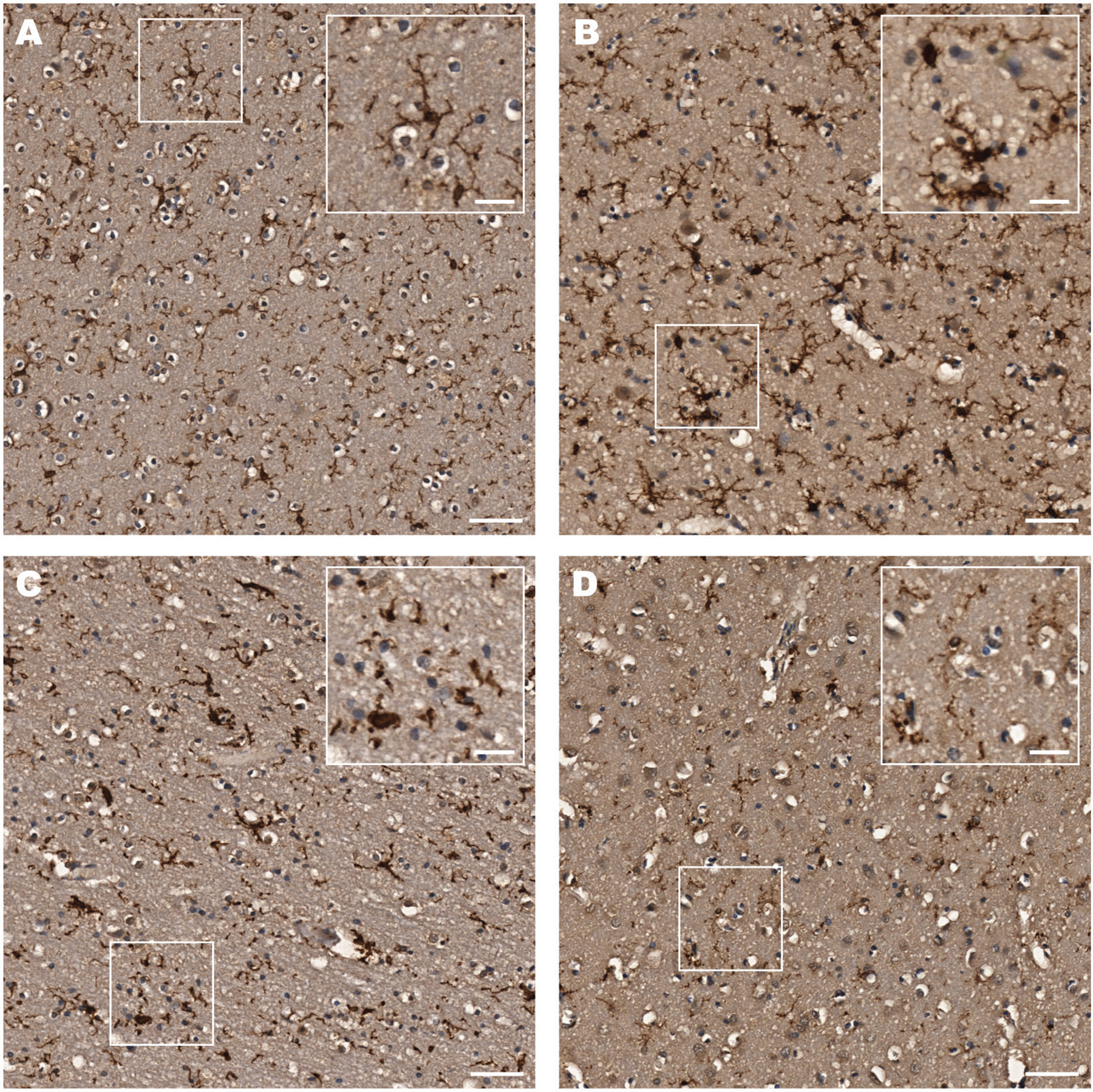
Microglial morphologies in the prefrontal cortical grey matter. A series of photomicrographs shows different microglial morphologies in the cortex of neurologically normal individuals vs those with AUD. (A) Normal (ramified) microglia in the prefrontal cortical grey matter of a 60-year-old control (C1). (B) Activated microglial prefrontal cortical grey matter of a 54-year-old AUD case (A4). (C) A mixed pattern of activated and dystrophic microglia in a 60-year-old AUD case (A3). (D) Relatively sparse and largely dystrophic microglia in a 51-year-old AUD case (A1). Scale bars: 50 μm (low resolution), 20 μm (high resolution).

Ramified, and only the occasional reactive, IBA1^+^ cell morphologies were frequently seen in controls, while reactive microglia were more common in AUD cases. Both cases and controls had dystrophic microglia with fragmented processes, especially cases A1 and A3. Dystrophic microglia were also more sparse in A1, the youngest patient (Figure 6). Importantly, a review of pathological data showed that all donors with dystrophic microglia had relatively low brain pH (Supplementary File 1).

### Astrocyte density

**Figure 7.**
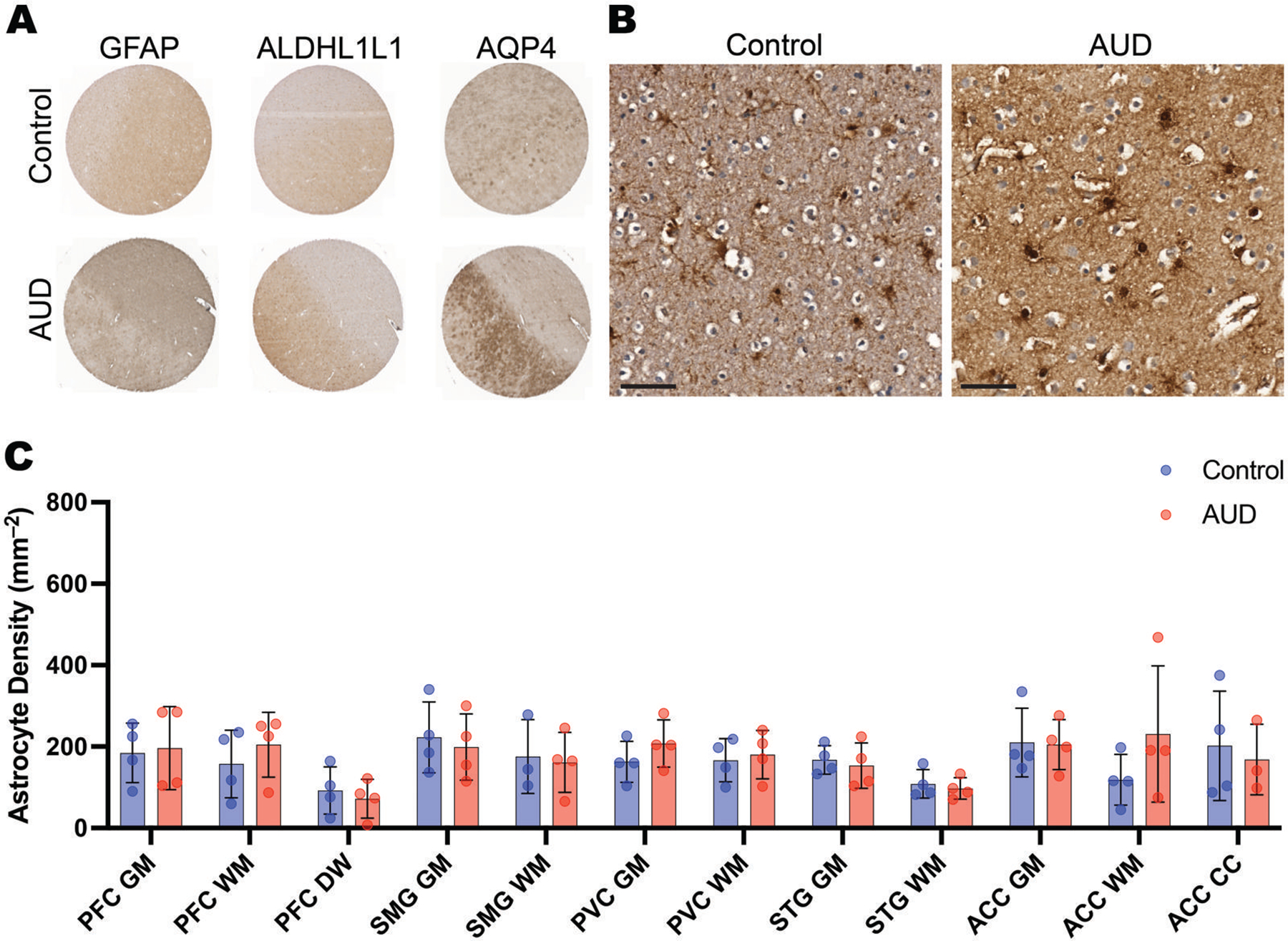
Astrocyte density. As part of a multiplex immunofluorescence protocol, astrocytes were stained for ALDHL1L1. (A) Typical AUD cases (lower row) show greater immunoreactivity than the controls (upper row) for all 3 astrocytic markers. (B) Higher resolution images show the apparent increased ALDHL1L staining in the PFC of an AUD case vs a control. (C) A histogram shows that astrocyte density in AUD cases (red) and controls (blue) across all cortical regions and both grey and white matter. Astrocytes were stained with ALDHL1L. Data shown as mean ± SD.

We tested the astrocytic markers GFAP, ALDH1L1, and AQP4 using both single-plex (DAB) (Figure 7A) and mIF. All 3 had previously been shown in our laboratory to demonstrate astrocytic features in human postmortem brain tissue. Out of the 3 markers, ALDH1L1 was the best marker for assessing density using automated image analysis tools because of its expression in the cell body only (Figure 7B). There was no difference in either the mean cortical density of ALDHL1L-positive cells between AUD cases (mean = 173 ± 85 per mm^2^) and controls (164 ± 76 per mm^2^), or in any individual region (Figure 7C)

### Astrocyte size

**Figure 8.**
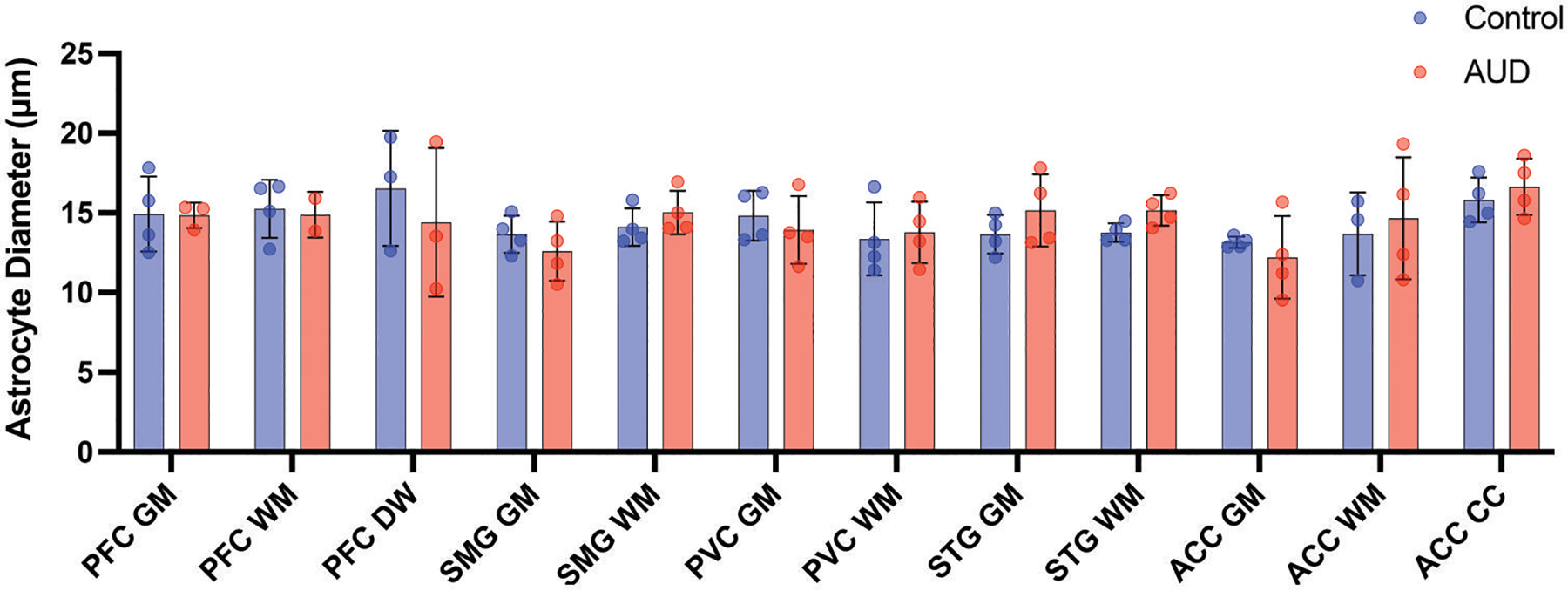
Astrocyte size. A histogram shows that astrocyte cell body size in AUD cases (red) was like controls (blue) across all cortical regions and both grey and white matter. Scale bars = 50 μm. Data shown as mean ± SD.

In normal brain tissue, only astrocytic nuclei are visible with histological stains like hematoxylin and eosin. In contrast, when the brain is injured, astrocytes hypertrophy, and their cell bodies and processes are clearly visible. Therefore, cell body diameter can be used as a proxy for activation status. Given the increased signal-to-noise ratio, measurements were carried out on ALDHL1L from mIF images. There was no regional or global difference in mean astrocyte cell body diameter between AUD cases (mean = 14.41 ± 2.40 μm) and controls (14.36 ± 1.85 μm; P = .85; Figure 8).

### Other astrocyte markers

**Figure 9.**
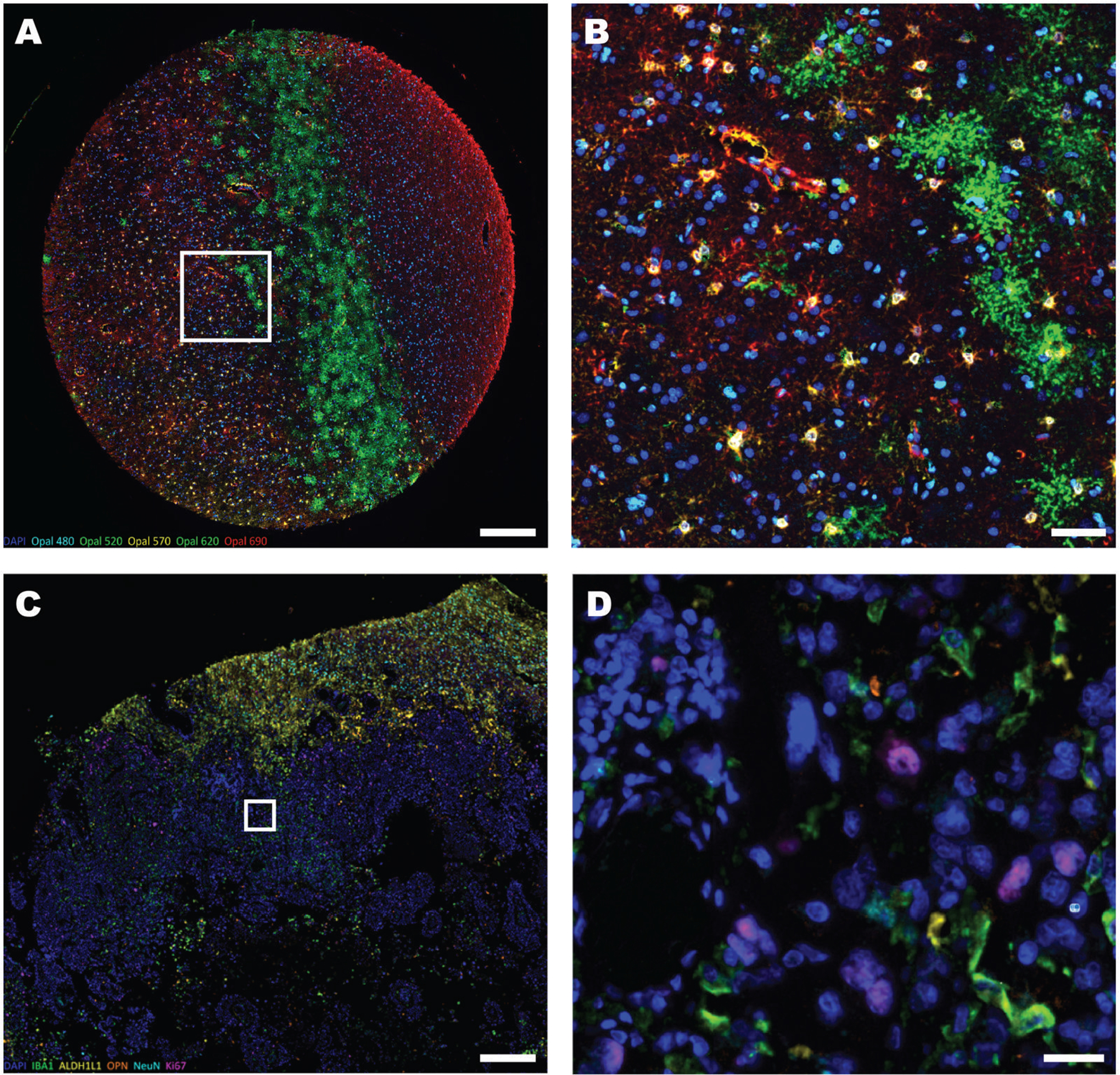
Investigation of astrocytic proliferation in AUD. (A) A multiplex immunofluorescent image from the PVC from an AUD case (A1) shows 3 different astrocytic markers, GFAP (red), ALDH1L1 (yellow), and AQP4 (green) along with the oligodendrocyte marker, ASPA (light blue), the microglial marker, Iba1 (pink) the nuclear stain DAPI (dark blue), and the proliferative marker, Ki-67 (white). (B) Higher resolution image of (A) showing the distinctive staining of astrocytic processes by AQP4 (green) with some colocalization with ALDH1L1 (yellow). Similarly, there is colocalization between GFAP (red) and ALDH1L1 but rarely all 3 markers together. (C) A multiplex immunofluorescent image demonstrates Ki-67-positive nuclei (pink) nuclei in a glioblastoma sample in the cerebrum with Iba1 (green), ALDH1L1 (yellow), NeuN (light blue), and the inflammatory microglial marker osteopontin (OPN, orange). The tumor shows dense DAPI (dark blue) staining in the center of the image, adjacent to the NeuN and ALDH1L1 staining of normal brain tissue in the upper left region. (D) A high-resolution image of C shows Ki67-positive mitotic figures (pink) within the highly cellular tumor along with activated microglia (green). Scale bars: *A* = 300 μm, *B* = 45 μm, *C* = 450 μm, *D* = 30 μm.

The astrocyte markers GFAP and AQP4 stain the full complex, branched structure of these “tree-like” cells.^29^ Unlike ALDHL1L staining, it was not possible to accurately quantify either marker using our automated image analysis pipeline. Subjectively, there are no obvious differences in GFAP staining between AUD cases and controls, but AQP4 staining at the GM-WM boundary appeared denser in AUD cases (Figure 9A and B). mIF showed colocalization between ALDH1L1 and AQP4-positive cells and between GFAP and ALDH1L1-positive cells but rarely were all 3 markers found together. We also added the proliferative marker Ki-67 to the mIF protocol for 5 of 8 TMAs and, as expected, there was no co-localization with the 3 astrocyte markers, ASPA or IBA1 (Figure 9A and B). As a positive control for cell proliferation, we applied this same protocol to tissue from a patient with glioblastoma. In contrast, Ki-67-positive nuclei (and mitotic bodies) were clearly visible within the tumor (Figure 9C and D).

### Cell densities in whole-section images vs TMAs

**Figure 10.**
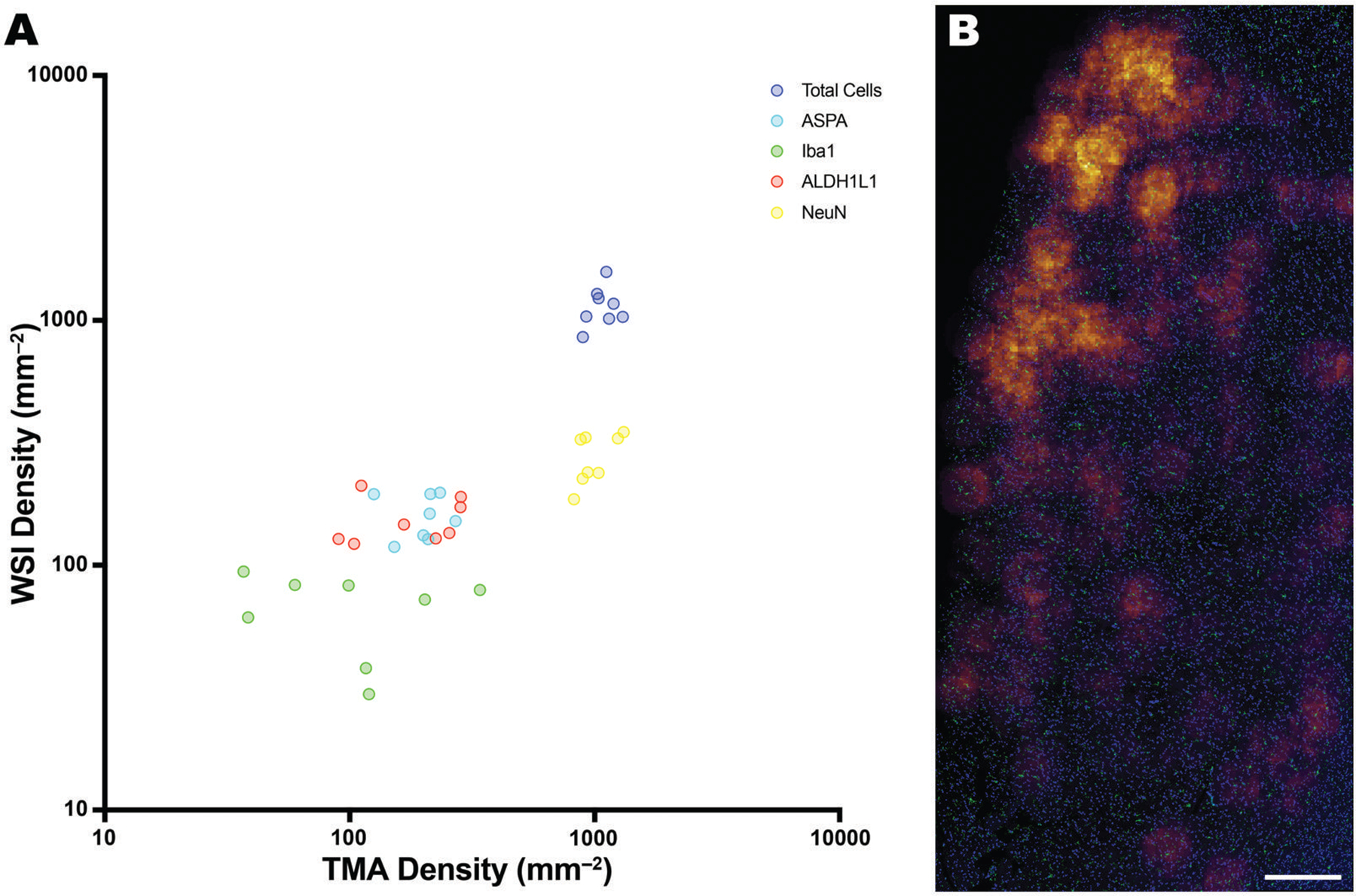
Comparison of TMA counts with WSIs. (A) A scatter plot showing the alignment between TMA and WSI counts of the PFC GM for total cells (dark blue), ASPA (light blue), Iba1 (green), ALDH1L1 (red), and Neun (yellow). Globally, TMA and WSI counts were well correlated (*r* = 0.8368; *P* < .0001). (B) A heat map created using QuPath, from a WSI of the PFC GM, showing the heterogenous distribution of microglia in the human cortex. Areas of high occurrence are colored in orange, while areas of lower occurrence are colored in blue. Scale bar = 500 μm.

We typically use a stereological method to quantify cells from whole-section images (WSI) that we adapted from a manual counting method developed in our laboratory.^4^ We have also recently described disparities between cell counts from WSI and TMAs from AD cases and controls.^21^ Here, the composite cell counts between TMA vs WSI counts were well correlated (*r* = 0.8368; *P* < .0001; Figure 10A). There was insufficient data to test each cell type individually but visually, total cells, ALDH1L1-, and ASPA-positive cells were aligned between TMAs and WSIs. Notably, NeuN counts from TMAs were higher than those from WSIs. As TMA cores often excluded layers I and II of the cortices, while WSIs sampled all cortical layers, TMAs would contain a more concentrated sample of neurons. Microglial counts did show a visual dissimilarity on the scatter plot and thus probable mismatch between TMA and WSI counts. Microglia are distributed heterogeneously in tissue as demonstrated in by a heatmap analysis, so TMA sampling is less likely to capture the mean microglial density of a region (Figure 10B).

## DISCUSSION

TMAs are a high-throughput platform typically used for assessing biomarkers across large cohorts by way of miniaturizing and amplifying finite tissue resources.^19^ We have previously used this approach to quantify the hallmark pathologies of AD.^21^ In AD, we have shown that amyloid areal burdens and neurofibrillary tangles can be quantified by TMAs, but we lack hallmark pathologies in AUD. Therefore, we need to rely on changes in cell densities and morphologies. Here, we demonstrated that TMAs combined with mIF, and a semi-automated pipeline allowed for a high-throughput approach to pathology quantification in AUD brains.

We had previously described mild neuron loss in the PFC of uncomplicated AUD cases, and wider neuron loss in those with concurrent thiamine deficiency.^4^ However, in a later study, we failed to replicate the difference in neuronal density in the PFC of uncomplicated AUD cases compared to controls.^7^ Similarly, there were no differences in neurons between cases and controls in the current study. Neurons accounted for ~50% of total cells in all cortical regions other than the PVC (~60%), as per previous studies.^30,31^

The mean microglia density across all regions and donors was higher in AUD than controls. However, there was no difference in any individual region, although the one region (PFC GM) approached significance. At the very least, our results are consistent with a subtle increase in microglial activation in ARBD^32^ and a more comprehensive, stereologically-based quantification study of the PFC GM, is therefore warranted. A power analysis (openepi.com) suggests that a sample size of 29 AUD cases and 32 controls would be 80% powered to confirm the difference in mean microglial density seen here. Microglial proliferation is another possibility and we had previously described proliferation in some alcoholics with concomitant hepatic encephalopathy but not in “uncomplicated” alcoholics.^28^ Three of the 4 AUD cases (A2 being the exception) here had histological evidence of hepatic encephalopathy, that is, the presence of Alzheimer type 2 astrocytes, but none displayed co-localization between the proliferative marker, Ki-67 and IBA1. When cell counts were compared to clinical data, significant correlations were seen (Supplementary File 3). Higher lifetime alcohol intake did, for the most part, positively correlate with higher microglial counts.

Assuming AUD is characterized by microglial activation, single-cell transcriptomics should then be very useful in elucidating the exact microglial subtype involved. In the largest study to date in AUD (*n* = 36 vs 37 controls) microglia could be divided into 2 major subpopulations, homeostatic and disease-associated.^33^ The latter could be further subdivided into disease-associated microglia^34^ and interferon-responsive microglial subtypes,^35^ where the prevalence of the latter was correlated with total drinking years. Differential gene expression was also prominent in astrocytes with co-expression analysis suggesting an integrated response between the 2 glia types to chronic alcohol intoxication.

Given that pathological studies and imaging work have demonstrated wide-ranging WM atrophy and myelin abnormalities in ARBD, we hypothesized here that oligodendrocytes would be lost. To the best of our knowledge, this had not been previously investigated. Contrary to this idea, we found no differences in the density of oligodendrocytes or their structure in this study. One AUD case (A1) had a relatively high density of oligodendrocytes in their CC, but this pattern was not seen in other WM regions. A caveat here is that we did not have a marker for oligodendrocyte precursor cells but any anomaly in these cells would presumably be reflected in mature oligodendrocyte numbers.

Our laboratory has previously optimized 3 different astrocyte markers to show different features and, potentially, activation status^29^; all 3 were employed in the current study. GFAP and, particularly, AQP4, stain the complete and complex tree-like structure of astrocytes.^36,37^ Quantification via pixel counts was possible for these 2 markers, but there was no obvious way to normalize for cell density. ALDHL1L immunostaining, in contrast, was restricted to the cell body or soma. This allowed astrocyte density to be accurately assessed, and we could also use cell body size as a proxy for activation status. Neither of these 2 indices were different between AUD cases and controls.

AQP4, is an astrocyte-specific water channel and a key component of glymphatic solute transport^38,39^; it is known to augment cytotoxic brain edema.^40^ Altered astrocytic AQP4 polarization, after stimulation, might also be important in ARBD. AQP4 is located on astrocyte foot processes that abut against cerebral blood vessels and form the parenchymal component of the blood-brain-barrier (BBB). Consistent with this idea is an imaging study that showed that ARBD was associated with enlarged perivascular spaces.^41^ These spaces lie between the foot processes and endothelium within the BBB.^42^ Increased AQP4 reactivity has been reported in the spinal tract of the trigeminal nerve after acute ethanol exposure in a mouse model but not in the reticular nucleus or cerebellum.^43^ In the current study, AQP4 staining was prominent at the GM-WM boundary and appeared denser in the AUD cases. As discussed above, AQP4 is typically found adjacent to blood vessels and brain surfaces. However, deeper GM-WM boundary staining has also been described in normal brain^44,45^ and reported as modified in diseases such as multiple sclerosis.^46^ The latter study suggested these were WM fibrous astrocytes. The challenge now is to adapt our algorithms to accurately quantify markers such as AQP4, as complex staining patterns are the rule, rather than the exception in the central nervous system. The situation is the same for GFAP, albeit there were no noticeable differences between AUD cases and controls here.

Quantification of human brain tissue should ideally use a stereologically based approach.^47,48^ Our group previously derived an abridged stereological methods for manual cell counts from single WSI per subject^4^ and we have applied this approach to semi-automated neuron quantification in AUD studies.^7^ In a recent case-control study of AD using TMAs we noted a discrepancy between cell counts from TMAs and our previous work in WSIs.^49^ Here, the TMAs and WSI counts aligned for oligodendroglia, astrocytes, and total cell counts. NeuN TMA counts were consistently lower than that of the WSIs and we speculate that this is likely due to the inclusion of the cortical layers I and II in the WSIs. Microglial counts between TMAs and WSIs were dissimilar. This may reflect the heterogenous nature of microglial density across the brain.^50^ However, our results are consistent with previous reports of varying IBA1-positive microglia density in the human brain.^51^

Overall, our results indicate that a 2 mm core sampling TMA schema offers a powerful tool for the rapid assessment of brain-wide ARBD. There were no obvious changes in the classes of brain cells; this is consistent with our previous studies of “uncomplicated” alcoholics for neurons and microglia.^7,28^ Nonetheless, the study had several limitations. First and foremost, this was a “first pass” study of the techniques, and we deliberately chose a small cohort and only studied male subjects. We had previously applied this same approach to AD and concluded that TMAs give equivalent data to whole-section-based studies in terms of hallmark pathologies like plaques and tangles but could not fully recapitulate cell density findings.^21^ Therefore, this work needs to be validated using whole tissue sections in which the approach is close to exhaustive stereological studies, at least for neuron counts, at least.^4^ Second, this study was restricted to cerebrum but should be extended to subcortical regions such as the thalamus, brain stem, and cerebellum for completeness. Third, a sufficiently powered whole-section study must include both sexes given the established differential effects of alcohol on male and female brains.^52^ However, sex effects seen with neuroimaging are subtle and not consistent across cortical and subcortical regions.^52^ Hence, for neuropathological confirmation of these sex findings, there remains a trade-off between breadth and depth, and TMAs may still be the most practical option.

Finally, the sampling method used here, while commonly used in the field, is susceptible to systematic bias. The densities reported only reflect those seen in slim, 7-μm-thick tissue sections, and not the entire 3D composition of the tissue. Indeed, it might be more precise to refer to the quantification of cell profiles here rather than cell themselves. This limitation has been extensively illustrated in the literature.^18^ Moreover, stereological approaches may have yielded different results, although the comparison of PFC counts from both TMAs and WSIs shows a reasonable correlation for all cells other than microglia. Nonetheless, the approach used here remains a practicably viable and resource-efficient method for estimating cell densities.

Looking forward, both TMAs and WSI studies of human brain tissue can be further refined by including more cell subtype-specific markers. The use of mIF here showed that overlap between all 3 astrocyte markers was relatively uncommon. This is consistent with the idea of multiple astrocytic subtypes,^53,54^ and the potential differential vulnerability of subtypes in AUD, as shown in other neurodegenerative diseases.^55^ Subtypes of all major brain cells are being continually identified by single-cell technologies^56^ and the largest study to date in AUD suggests an interaction between microglial and astrocyte subtypes.^33^ In contrast, fluorophore-based techniques are still quite limited, but technologies such as the Phenocycler (Akoya Biosciences) are now able to multiplex more than 40 different targets.^57^ It is also possible that the neuropathological basis of ARBD lies at the subcellular level like neuronal synaptic density or the degree of gap junctional coupling between astrocytes. Exploring the latter requires an even greater “trade-off” between depth and breadth of sampling than the current work and will require further improvements in high-throughput, high-resolution imaging to uncover the true nature and extent of ARBD neuropathology.

## Supplementary Material

Supp file 1

Supp file 3

Supp file 2

Supplementary material is available at academic.oup.com/jnen.

## Figures and Tables

**Table 1.** Cohort demographics and characteristics.

Mean (SD)	AUD	Control	*P*-value
Age (years) (SD)	56 (5.8)	56 (5.2)	.97
Brain weight (g) (SD)	1392 (87)	1471 (100)	.69
Postmortem interval (h) (SD)	34 (12.5)	27 (6.1)^a^	.63
Brain pH (SD)	7 (0.3)	6 (0.14)	.43
Lifetime alcohol consumption (kg)	2617 (1138)	57 (63.4)^a^	.06
AUDIT-C	12 (0)	4 (3.8)	.03

a Data for 3 controls only.

## Data Availability

All data generated or analyzed during this study are included in this published article (and supplementary information files). There were no publicly archived datasets analyzed or generated during the study.
