# Supplementary figures and images for: Quantification of the neuropathology of alcohol use disorder using tissue microarrays
Source: J Neuropathol Exp Neurol. Author manuscript; Available in PMC 2026 Jun 5. (PMC13236575; doi:10.1093/jnen/nlaf147)

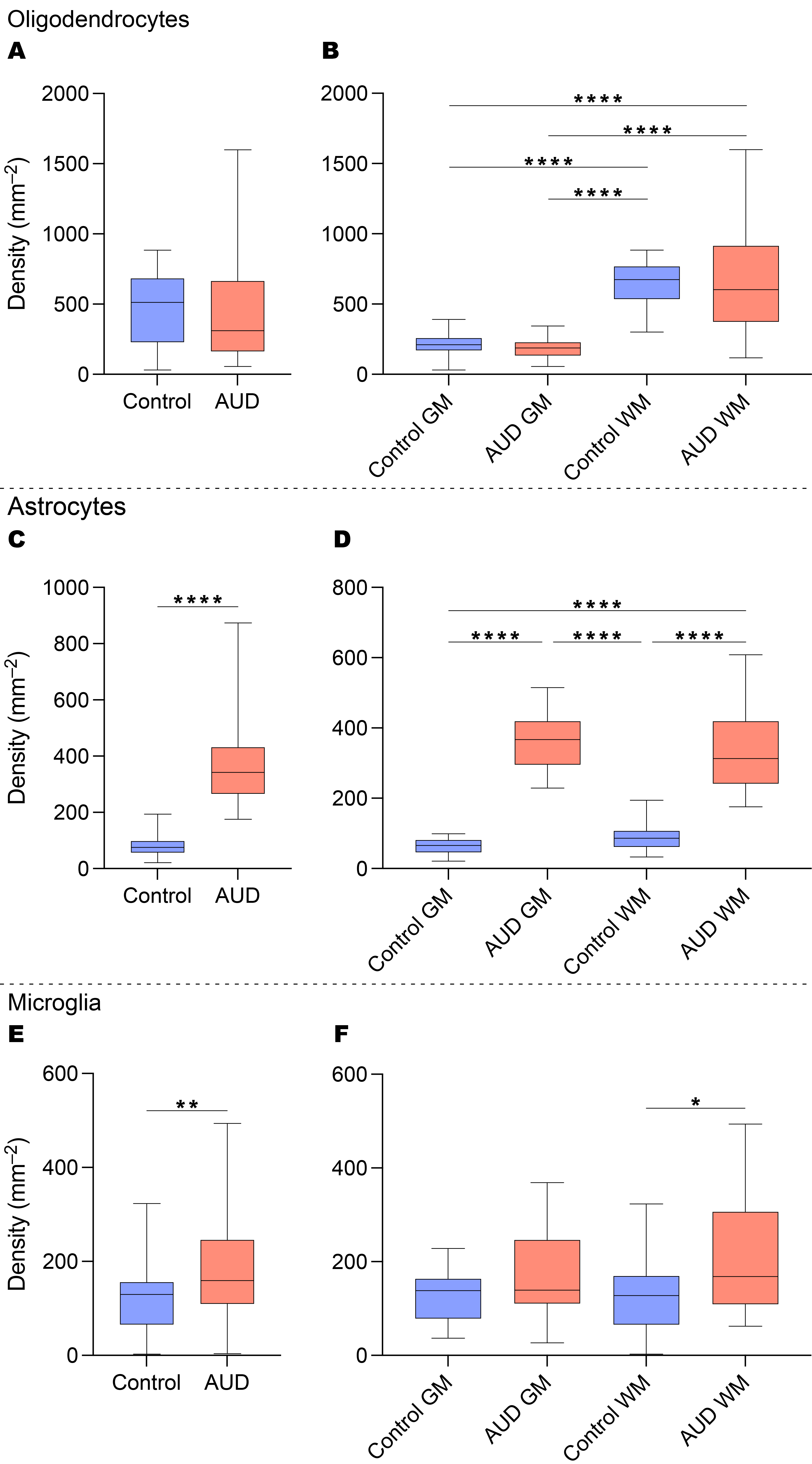

Supplement: Supp file 1 [file NIHMS2175100-supplement-Supp_file_1.png]

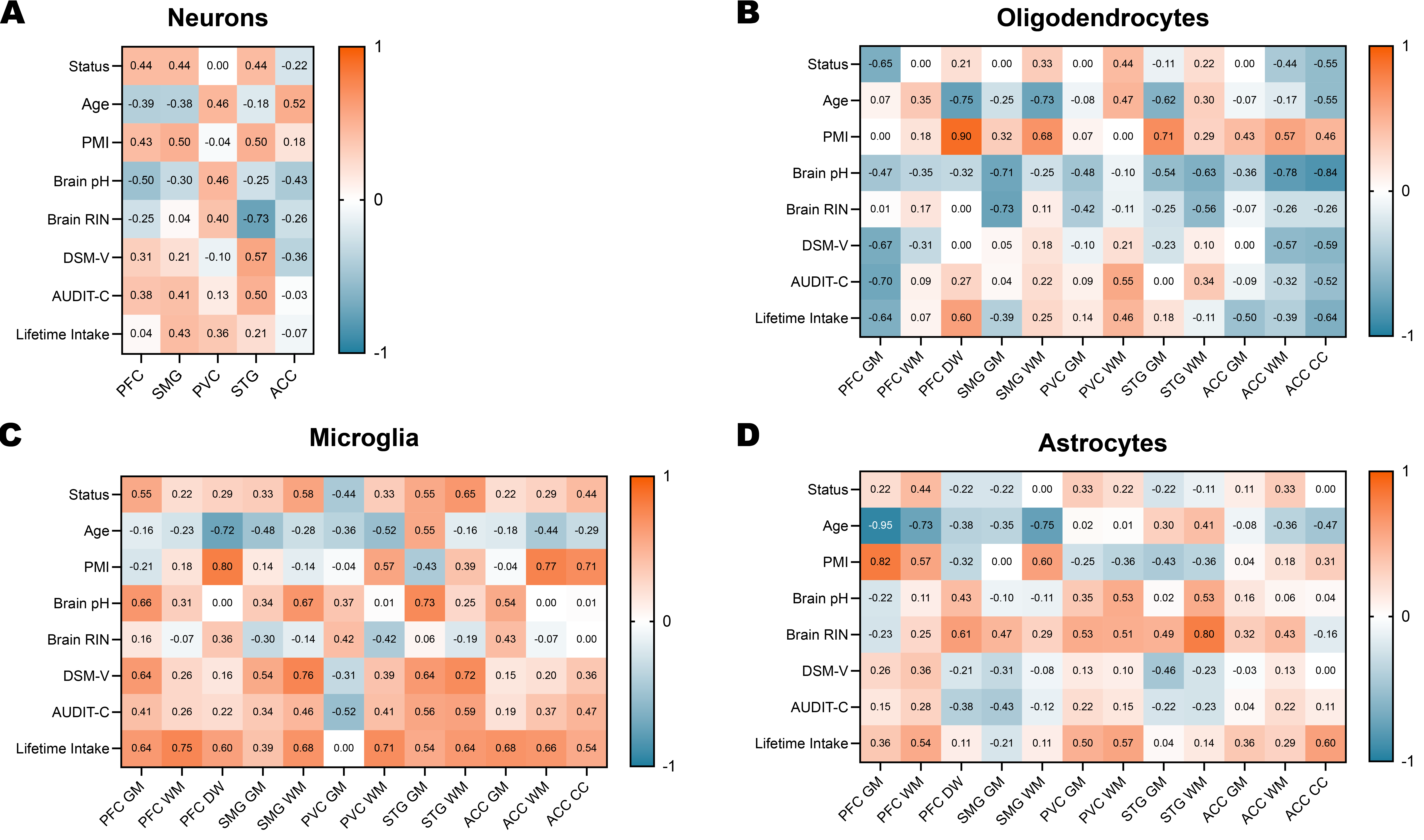

Supplement: Supp file 3 [file NIHMS2175100-supplement-Supp_file_3.png]
