# Supplementary material for: Quantification of the neuropathology of alcohol use disorder using tissue microarrays
Source: J Neuropathol Exp Neurol. Author manuscript; Available in PMC 2026 Jun 5. (PMC13236575; doi:10.1093/jnen/nlaf147)
Supplement: Supp file 2 [file NIHMS2175100-supplement-Supp_file_2.pdf]

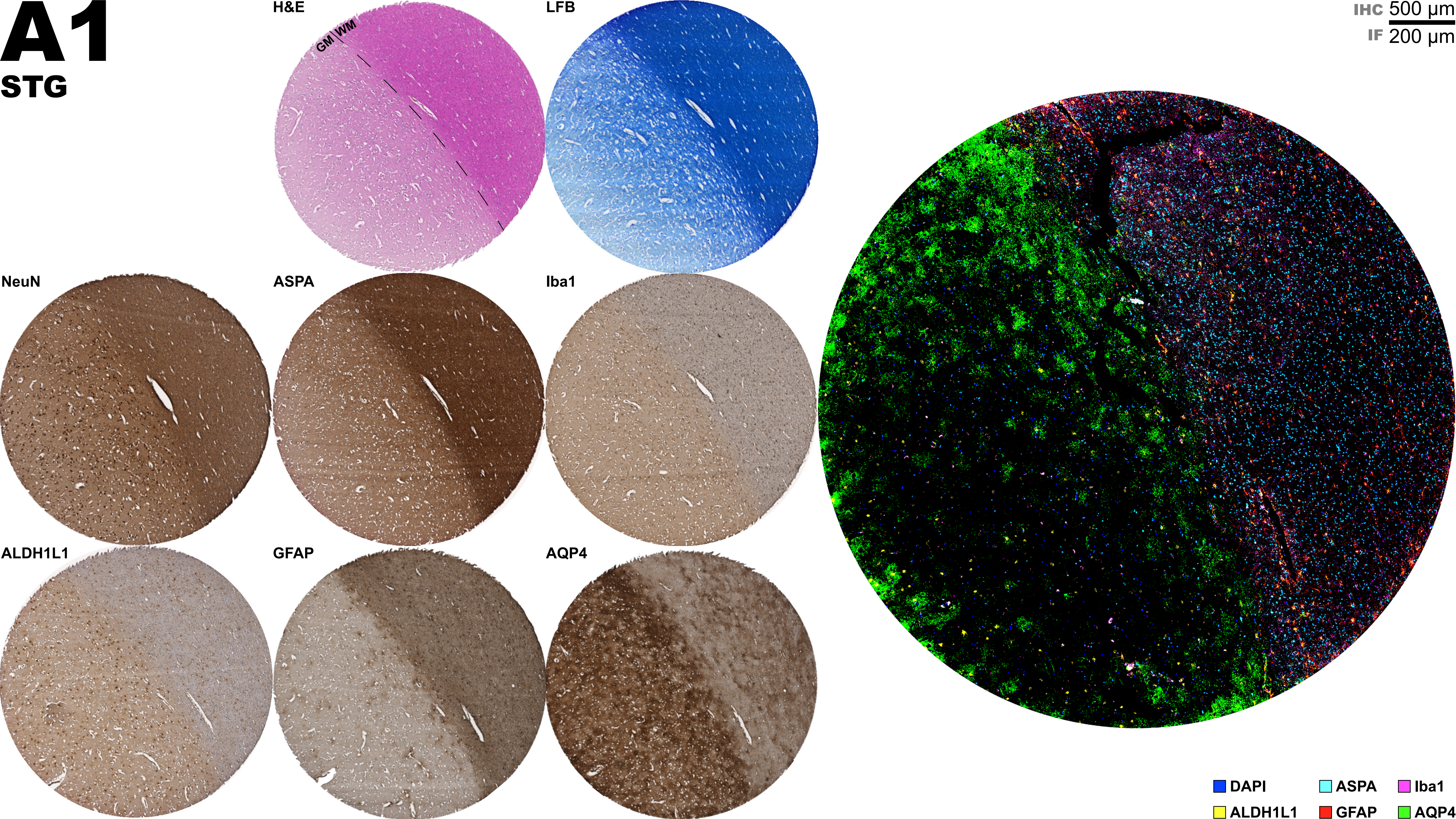
A1
STG
H&E
GM
WM
LFB
IHC 500 µm
IF 200 µm
NeuN
ASPA
Iba1
ALDH1L1
GFAP
AQP4
DAPI
ASPA
Iba1
ALDH1L1
GFAP
AQP4

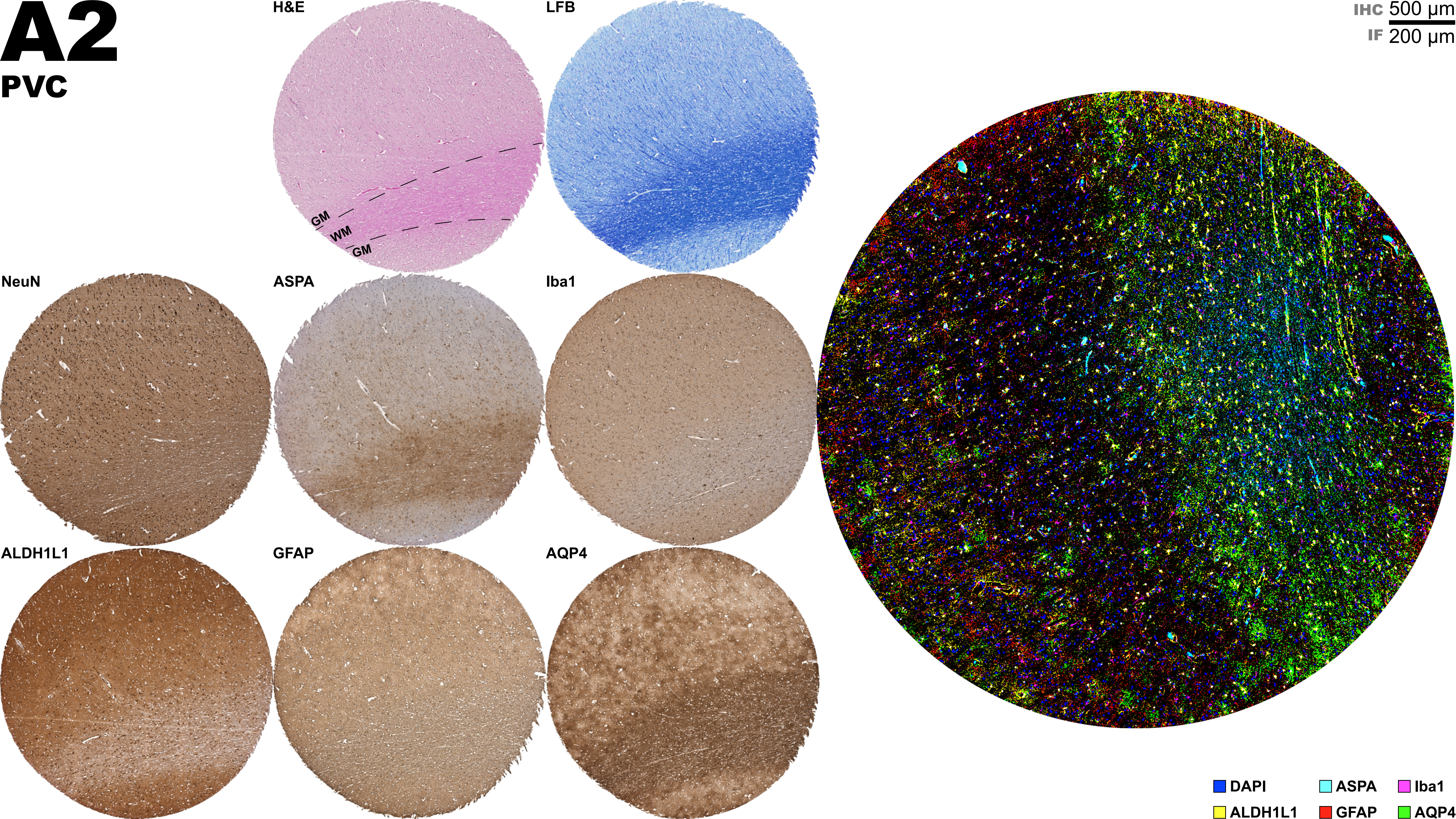
A2
PVC
H&E
LFB
IHC 500 µm
IF 200 µm
GM
WM
GM
NeuN
ASPA
Iba1
ALDH1L1
GFAP
AQP4
DAPI
ASPA
Iba1
ALDH1L1
GFAP
AQP4

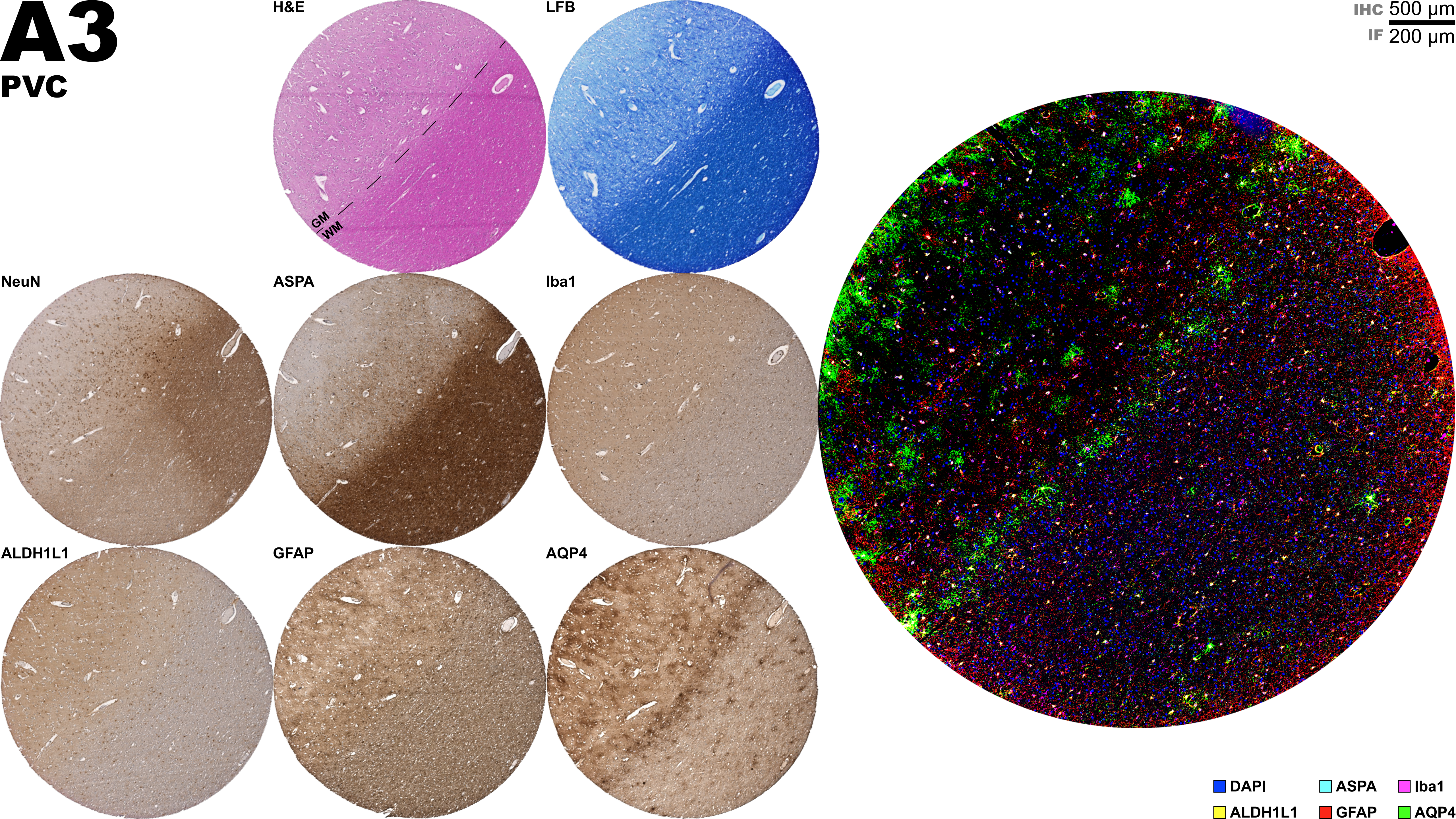

A3
PVC
H&E
LFB
GM
WM
NeuN
ASPA
Iba1
ALDH1L1
GFAP
AQP4
IHC 500 µm
IF 200 µm
DAPI
ASPA
Iba1
ALDH1L1
GFAP
AQP4

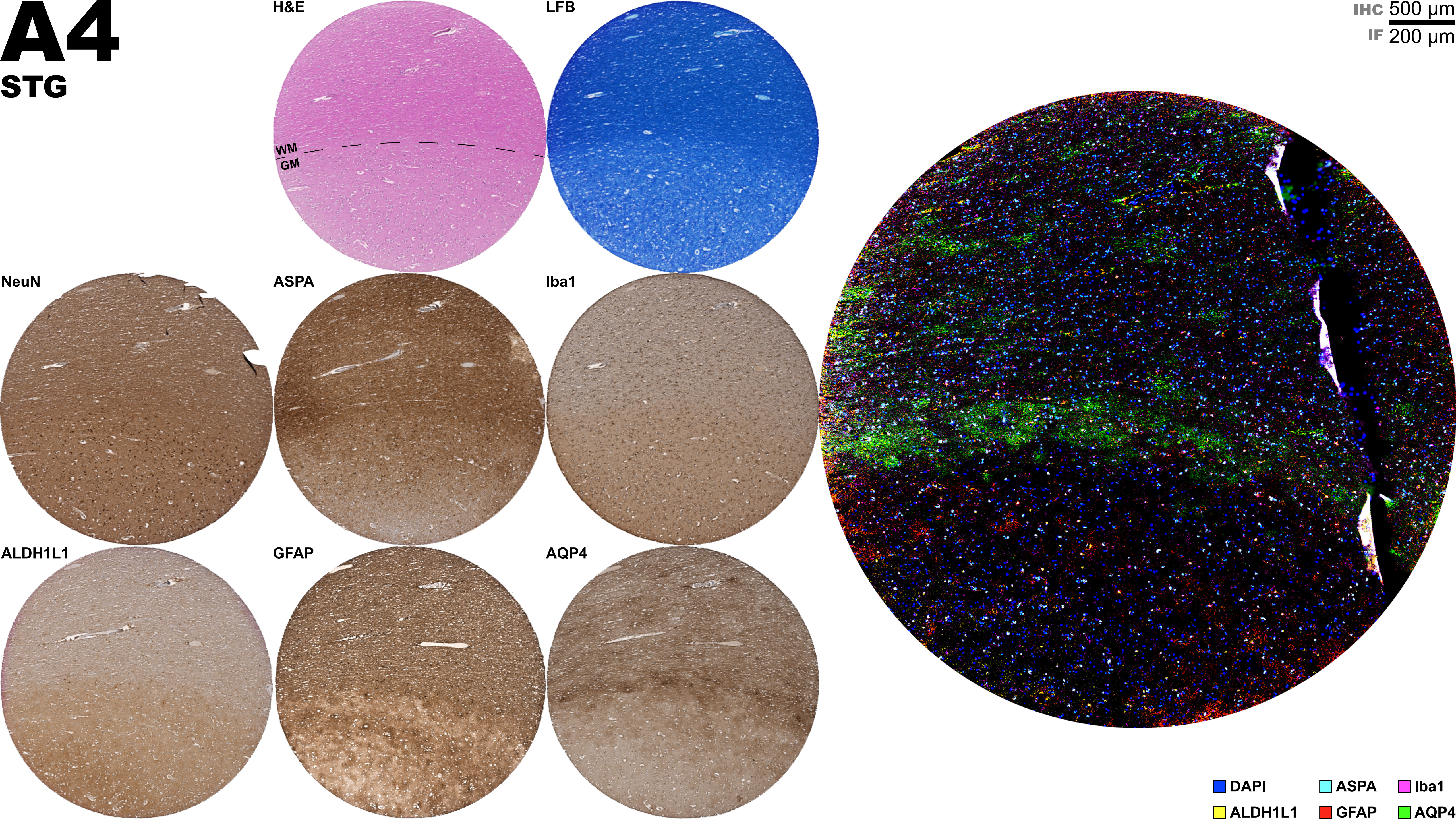
A4
STG
H&E
LFB
WM
GM
NeuN
ASPA
Iba1
ALDH1L1
GFAP
AQP4
IHC 500 µm
IF 200 µm
DAPI
ASPA
Iba1
ALDH1L1
GFAP
AQP4

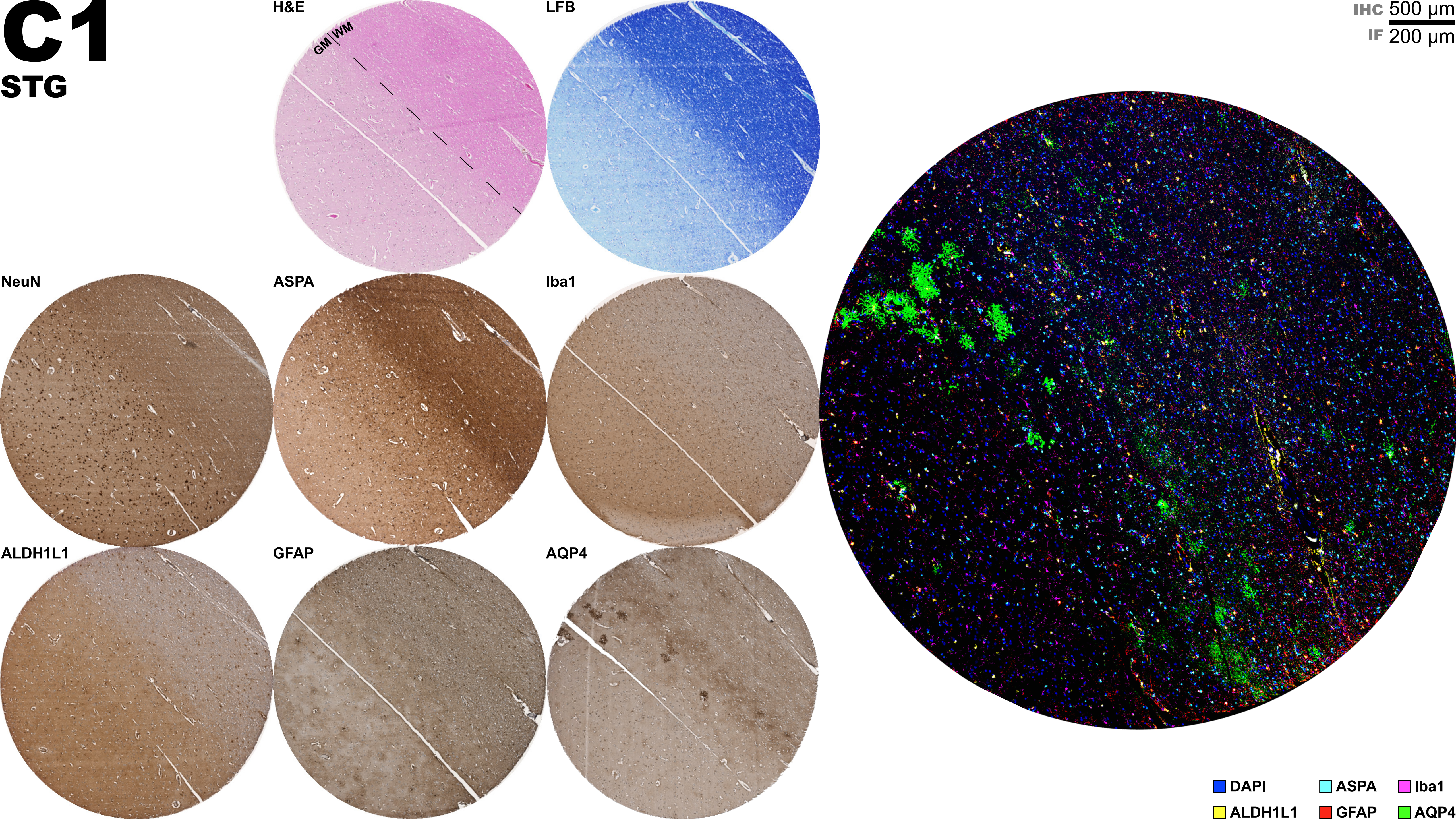
C1
STG
H&E
GM
WM
LFB
NeuN
ASPA
Iba1
ALDH1L1
GFAP
AQP4
IHC 500 µm
IF 200 µm
DAPI
ASPA
Iba1
ALDH1L1
GFAP
AQP4

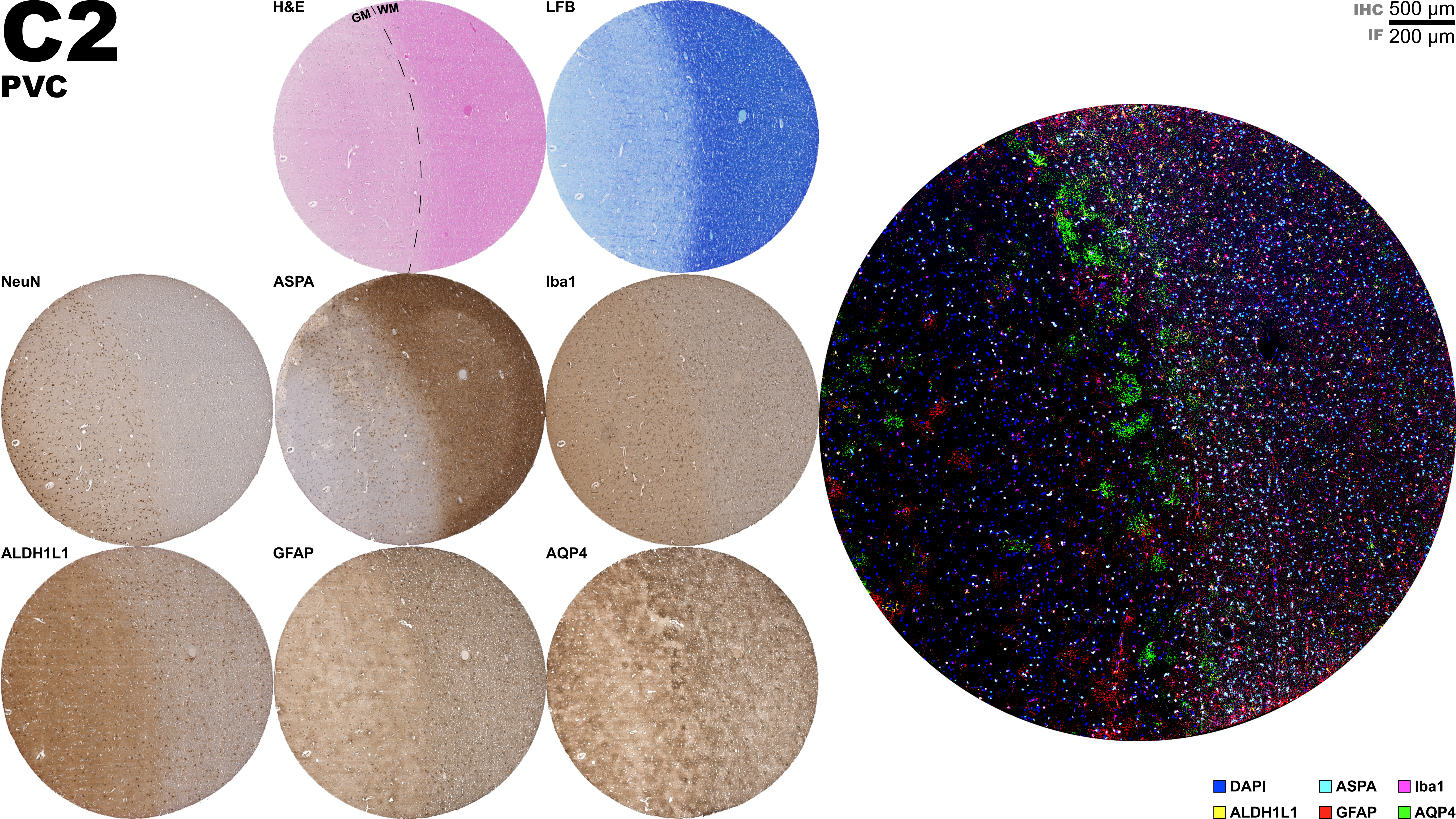
C2
PVC
H&E
GM
WM
LFB
NeuN
ASPA
Iba1
ALDH1L1
GFAP
AQP4
IHC 500 µm
IF 200 µm
DAPI
ASPA
Iba1
ALDH1L1
GFAP
AQP4

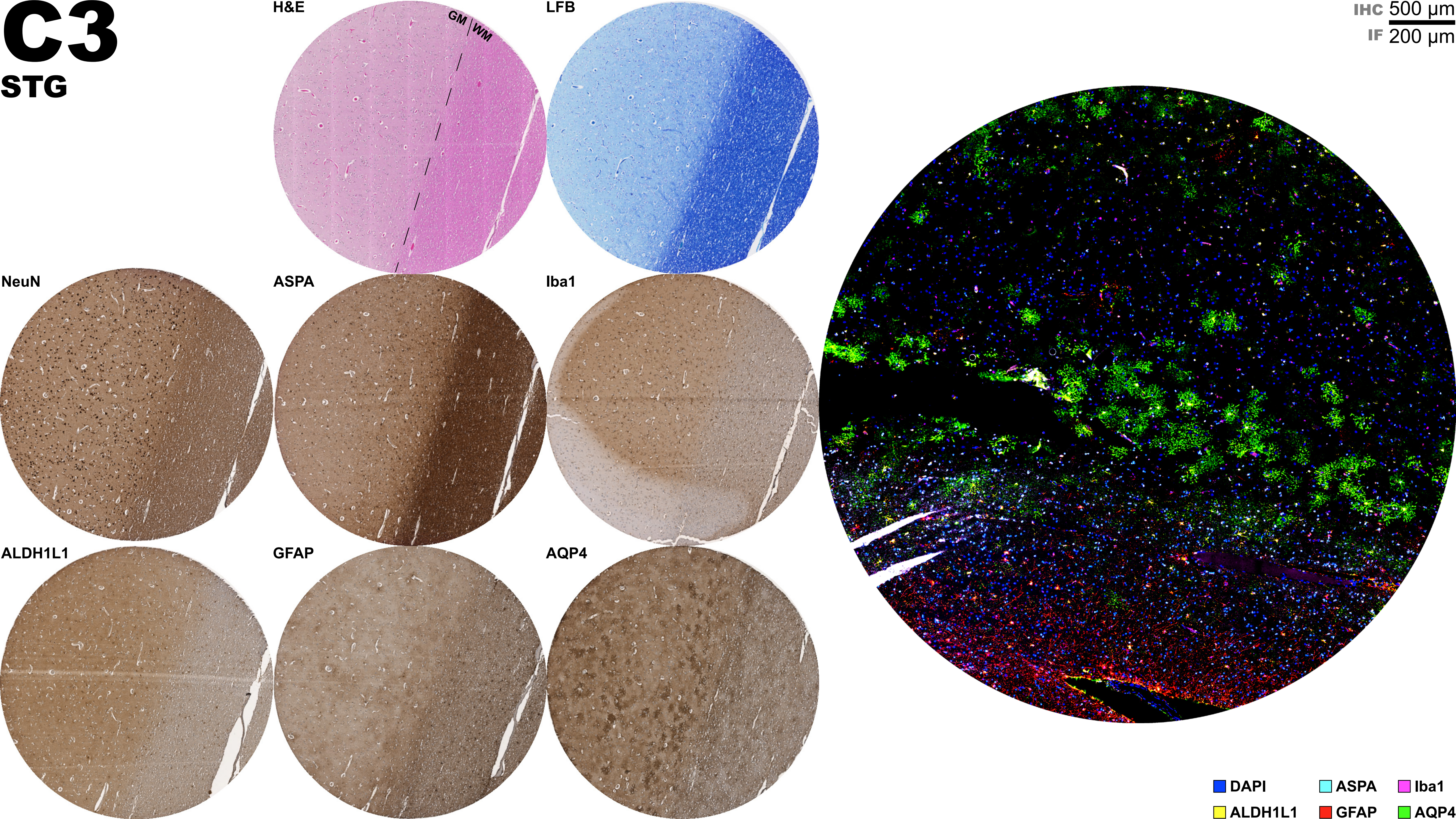
C3
STG
H&E
GM
WM
LFB
IHC 500 µm
IF 200 µm
NeuN
ASPA
Iba1
ALDH1L1
GFAP
AQP4
DAPI
ASPA
Iba1
ALDH1L1
GFAP
AQP4

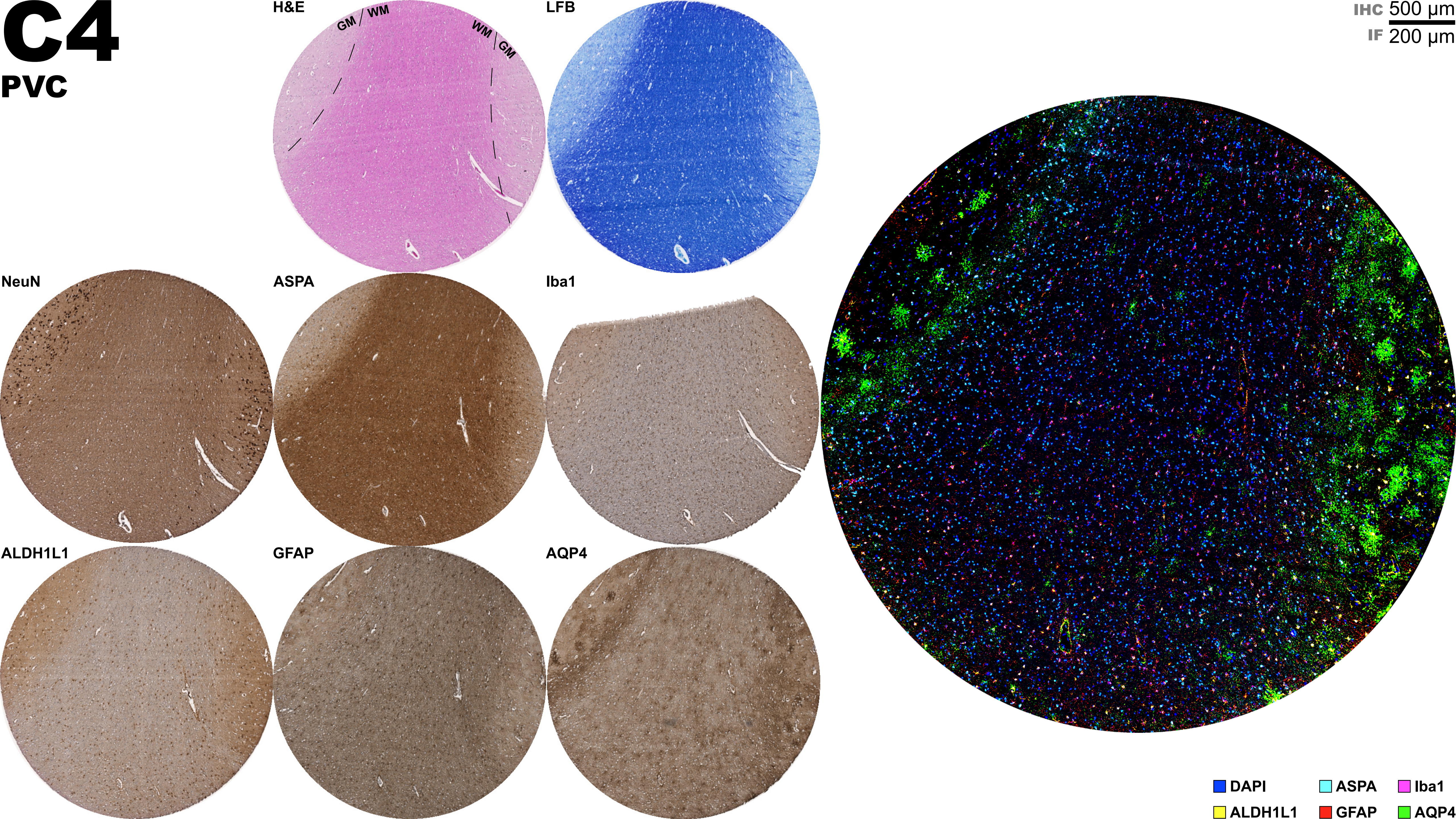
C4
PVC
H&E
GM
WM
WM
GM
LFB
IHC 500 µm
IF 200 µm
NeuN
ASPA
Iba1
ALDH1L1
GFAP
AQP4
DAPI
ASPA
Iba1
ALDH1L1
GFAP
AQP4
